# Age‐specific cardiovascular disease‐related mortality among patients with major gastrointestinal cancers: A SEER population‐based study

**DOI:** 10.1002/cam4.6305

**Published:** 2023-06-30

**Authors:** Gen Liu, Bo‐fang Zhang

**Affiliations:** ^1^ Department of Cardiology Renmin Hospital of Wuhan University; Cardiovascular Research Institute, Wuhan University; Hubei Key Laboratory of Cardiology Wuhan China

**Keywords:** age, cardiovascular disease, major gastrointestinal cancers, mortality

## Abstract

**Background:**

Studies have reported age as a risk factor for cardiovascular disease (CVD)‐related mortality; however, only a few studies have focused on the relationship between age and CVD‐related mortality, especially among major gastrointestinal cancers.

**Method:**

The present retrospective cohort enrolled patients with colorectal, pancreatic, hepatocellular, gastric, and esophageal cancer between 2000 to 2015 from the Surveillance, Epidemiology and End Results Registry (SEER). Standardized mortality ratio (SMR), competing risk regression, and restricted cubic spline (RCS) analyses were used in our study.

**Results:**

We analyzed 576,713 patients with major gastrointestinal cancers (327,800 patients with colorectal cancer, 93,310 with pancreatic cancer, 69,757 with hepatocellular cancer, 52,024 with gastric cancer, and 33,822 with esophageal cancer). Overall, CVD‐related mortality gradually decreased every year, and the majority were older patients. All cancer patients had a higher CVD‐related mortality rate than the general U.S. population. The adjusted sub‐hazard ratios for middle‐aged with colorectal cancer, pancreatic cancer, hepatocellular cancer, gastric cancer, and esophageal cancer were 2.55 (95% CI: 2.15–3.03), 1.77 (95% CI: 1.06–2.97), 2.64 (95% CI: 1.60–4.36), 2.15 (95% CI: 1.32–3.51), and 2.28 (95% CI: 1.17–4.44), respectively. The adjusted sub‐hazard ratios for older patients with colorectal cancer, pancreatic cancer, hepatocellular cancer, gastric cancer, and esophageal cancer were 11.23 (95% CI: 9.50–13.27), 4.05 (95% CI: 2.46–6.66), 4.47 (95% CI: 2.72–7.35), 7.16 (95% CI: 4.49–11.41), and 4.40 (95% CI: 2.28–8.48), respectively. A non‐linear relationship between age at diagnosis and CVD‐related mortality was found in colorectal cancer, pancreatic cancer, and esophageal cancer; their reference ages were 67, 69, and 66 years old, respectively.

**Conclusion:**

This study demonstrated that age was a risk factor for CVD‐related mortality among major gastrointestinal cancers.

## 1 INTRODUCTION

Gastrointestinal cancers are an umbrella term for cancer originating from the stomach, esophagus, pancreas, hepatobiliary system, large intestine (colorectal and anal regions), and small intestine,^1^, ^2^ with an estimated number of new cases in 2020 of 5,026,243, accounted for 27.8% of the global incidence of cancer and 35.8% of all cancer‐related deaths in 2020.^3^ By 2040, it is estimated that 8,051,490 new cases will be (27.9% of the global incidence of cancer), and 5,909,501 deaths (36.5% of all cancer‐related deaths).^4^

In the past two decades, there has been an improved clinical outcome for most cancer with the advancement of early diagnosis and treatment.^5^ The increased life expectancy of cancer survivors has also led to a higher occurrence of cardiovascular diseases (CVD).^5^ Among cancer survivors who had survived at least 5 years after diagnosis, the reported risk of CVD, such as hypertension, diabetes mellitus, and dyslipidemia, had a 1.7–18.5‐fold increase when compared with age‐matched counterparts with no cancer population, and the risk of CVD‐related mortality had a 1.3–3.6‐fold increase.^6^ This phenomenon may be due to the side effects of cancer therapies, such as chemotherapy, radiation therapy, and target immunotherapy, on the cardiovascular system.^7^, ^8^, ^9^ Another reason is that cancer and CVD have overlapping risk factors, such as tobacco use, obesity, and age.^10^, ^11^, ^12^ Notably, older people are more susceptible to CVD and frequent forms of cancer, which may be related to increased susceptibility caused by the mechanisms of biological aging.^13^ Many researchers have been treating age as a risk factor for CVD among cancer patients. For instance, Jeon et al. demonstraeted that patients with aged ≥50 years were more susceptible to CVD than younger patients (aged <50 years) with breast cancer who received chemotherapy.^14^ Ramai et al. reported that increasing age was associated with a higher likelihood of experiencing cardiac mortality in patients with major gastrointestinal cancers.^15^ However, these studies have not thoroughly demonstrated the relationship between age and CVD‐related mortality. The related literature is still limited on the relationship between age and CVD among the cancer population, especially for major gastrointestinal cancers. Hence, the present study explored (i) the distribution of age and its association with baseline characteristics, (ii) the standard mortality ratio (SMR) compared with the general U.S. population, and (iii) the association between age and CVD‐related mortality among major gastrointestinal cancers.

## 2 METHODS

### 2.1 Study participants

**FIGURE 1:**
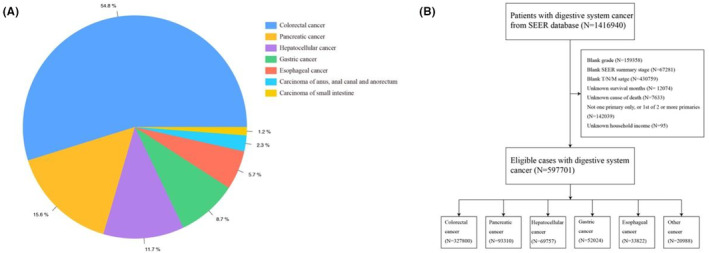
(A) The pie chart of gastrointestinal cancers distribution. (B) The selection flow chart of patients diagnosed with major gastrointestinal cancers in our study.

Our study is a retrospective cohort study. Data from the Surveillance, Epidemiology and End Results Registry (SEER) 17 registries from 2004 to 2015 and histologically verified as major gastrointestinal cancers: colorectal cancer, pancreatic cancer, hepatocellular cancer, gastric cancer, and esophageal cancer, were eligible for our study (Figure 1A). The demographic variables of interest were chosen using the SEER*Stat (version 8.4.0.1) software, which included age at diagnosis, sex, race, grade, SEER summary stage, T stage, N stage, M stage, treatment, marital status, and income. Figure 1B provides additional information on the inclusion and exclusion criteria for the final cohort of patients with major gastrointestinal cancers. A total of 576,713 patients with major gastrointestinal cancers were preserved in the final analyses. Institutional review board approval and informed consent were exempted because of the deidentified and publicly available characteristics of the SEER database. This cohort study was in accordance with the Strengthening the Reporting of Observational Studies in Epidemiology (STROBE) reporting guideline for observational studies.

### 2.2 Outcomes of interest

The primary outcome of our study was CVD‐related mortality among patients with major gastrointestinal cancers. The detailed CVD list in the SEER 17 registries database includes diseases of heart, cerebrovascular diseases, hypertension without heart disease, atherosclerosis, other diseases of arteries, arterioles, capillaries, and aortic aneurysm and dissection.

### 2.3 Statistical analysis

In our study, participants were divided into three groups based on their age at diagnosis (≤44, 45–59, and ≥60 years). The baseline characteristics were summarized using percentages for categorical variables and medians with interquartile ranges for continuous variables. The chi‐square test was used to evaluate intragroup differences in enumeration data, whereas the Kruskal–Wallis test was used to evaluate intragroup differences in measurement data.

Our study compared age‐specific CVD‐related mortality among major gastrointestinal cancers in the SEER data to the U.S. population from 2000 to 2019, termed the standardized mortality ratio (SMR). Age‐specific CVD‐related mortality from 2000 to 2019 was derived from the Centers for Disease Control and Prevention Wide‐ranging Online Data for Epidemiologic Research (WONDER) Tool. The number of expected deaths for each age category was the product of the number of cancer cases in each age stratum and age‐specific crude risk of CVD in the U.S. population. The SMR equaled the observed deaths over expected deaths. The 95% confidence interval (CI) of SMR was calculated using the Boice–Monson method.

As previously reported, the sub‐hazard ratio (SHR) was determined to be similar to the hazard ratio, but taking into consideration the hazard of competing events, which was suitable for exploring CVD‐related mortality among cancer patients.^16^, ^17^ In our study, competing risk regression was used to evaluate the association between age at diagnosis and CVD‐related mortality among major gastrointestinal cancers. Patients who died from cancer were regarded as competing events. Fine and Gary's competing risk regression analysis was used for assessing the initial predictors for CVD‐related mortality and estimating the cumulative incidence curve of CVD development. Those variables with 2‐sided *p < 0.05* in the univariate competing risk regression analysis were included in the multivariate competing risk regression analysis. In colorectal cancer, pancreatic cancer, hepatocellular cancer, and gastric cancer, variables of race, grade, SEER summary stage, age, T stage, N stage, M stage, treatment, and marital status were included in the multivariate competing risk regression. In esophagus cancer patients, we excluded the T stage variable to avoid the nonsymmetric or highly singular variance matrix.

Restricted cubic spline (RCS) analyses, based on the Cox proportional hazards regression model, were applied to depict the dose–response association and to explore the potential linear or non‐linear relationship of age with CVD‐related mortality, in which unadjusted or adjusted RCS analyses were conducted, respectively. To balance best fit and overfitting in the main splines for CVD‐related mortality, we selected the number of knots—between three and seven—according to the value of the Akaike information criterion (AIC); the lowest value and the smallest number of knots regarded as the optimal choice. Those variables adjusted for the RCS model were kept in line with those included in the multivariate Cox proportional hazards regression among various cancer types.

The competing risk regression and cumulative incidence curve of CVD‐related mortality were conducted using STATA/SE version 15.0. Other statistical analyses were performed using R, version 4.0.5. All tests were 2‐sided, and *p < 0.05* was regarded as statistically significant.

## 3 RESULTS

### 3.1 Characteristics of participants

In the primary SEER cohort, 597,701 patients with gastrointestinal cancers were identified. The top five types of gastrointestinal cancers (Figure 1A and Table S1) were 327,800 (54.8%) colorectal cancer, 93,310 (15.6%) pancreatic cancer, 69,757 (11.7%) hepatocellular cancer, 52,024 (8.7%) gastric cancer, and 33,822 (5.7%) esophageal cancer. These 576,713 patients were included in the final analyses.

In colorectal cancer and pancreatic cancer, there was an almost equal ratio of males to females (Table 1), but in hepatocellular cancer, gastric cancer, and esophageal cancer, males were the majority. In all five gastrointestinal cancers, whites were the dominant group. More than 60% of colorectal cancer patients were in the grade of moderately differentiated (Grade II), more than 65% of pancreatic cancer or hepatocellular cancer patients were of unknown grade, and over half of gastric cancer and over 40% of esophageal cancer patients were poorly differentiated (Grade III). Furthermore, 37.8% and 36.5% of colorectal cancer patients were in the localized and regional stage, respectively; 52.9% of pancreatic cancer patients in the distant stage; 43.1% of hepatocellular cancer patients in the localized stage; 30.0% and 38.1% of gastric cancer patients in the regional and distant stage, respectively; 31.4% and 38.2% of esophageal cancer patients in the regional and distant stage, respectively. Meanwhile, 44.1% of colorectal cancer and 33.5% of pancreatic cancer patients were diagnosed at the T3 stage, respectively; 35.2% of hepatocellular cancer patients at the T1 stage; 26.9% of gastric cancer patients at the T2 stage; 26.7% and 26.9% of esophageal cancer patients at the T1 and T3 stage, respectively. Lymph node involvement could be observed in 36.2% of colorectal cancer patients, 30.1% of pancreatic cancer patients, 8.3% of hepatocellular cancer patients, 41.8% of gastric cancer patients, and 44.5% of esophageal cancer patients. Patients with distal metastasis accounted for 20.3% of colorectal cancer, 49.4% of pancreatic cancer, 17.2% of hepatocellular cancer, 36.3% of gastric cancer, and 34.2% of esophageal cancer. In cancer treatment, more than 50% of colorectal cancer and 21.5% of gastric cancer patients suffered from surgery; 47.0% of pancreatic cancer patients and 46.0% of hepatocellular cancer patients received no/unknown therapy; and 31.0% of esophageal cancer patients received combined radiation and chemotherapy. Furthermore, most patients were married, half earned more than $64,999, while the other half earned less. Irrespective of the variable category, most patients aged ≥60 years at diagnoses.

**TABLE 1:** Baseline characteristics.

Variable	Colorectal cancer					Pancreatic cancer					Hepatocellular cancer					Gastric cancer					Esophageal cancer				
	Overall	≤44	45–59	≥60	*p* value	Overall	≤44	45–59	≥60	*p* value	Overall	≤44	45–59	≥60	*p* value	Overall	≤44	45–59	≥60	*p* value	Overall	≤44	45–59	≥60	*p* value
	*n* = 327,800	*n* = 20,003	*n* = 86,387	*n* = 221,410		*n* = 93,310	*n* = 2633	*n* = 19,342	*n* = 71,335		*n* = 69,757	*n* = 2511	*n* = 24,837	*n* = 42,409		*n* = 52,024	*n* = 3589	*n* = 12,032	*n* = 36,403		*n* = 33,822	*n* = 981	*n* = 8856	*n* = 23,985	
Sex (%)																									
Female	157,783 (48.1)	9534 (47.7)	37,042 (42.9)	111,207 (50.2)	<0.001	46,561 (49.9)	1222 (46.4)	7901 (40.8)	37,438 (52.5)	<0.001	18,896 (27.1)	778 (31.0)	4510 (18.2)	13,608 (32.1)	<0.001	19,519 (37.5)	1651 (46.0)	3912 (32.5)	13,956 (38.3)	<0.001	7106 (21.0)	176 (17.9)	1471 (16.6)	5459 (22.8)	<0.001
Male	170,017 (51.9)	10,469 (52.3)	49,345 (57.1)	110,203 (49.8)		46,749 (50.1)	1411 (53.6)	11,441 (59.2)	33,897 (47.5)		50,861 (72.9)	1733 (69.0)	20,327 (81.8)	28,801 (67.9)		32,505 (62.5)	1938 (54.0)	8120 (67.5)	22,447 (61.7)		26,716 (79.0)	805 (82.1)	7385 (83.4)	18,526 (77.2)	
Race (%)																									
White	259,140 (79.1)	14,948 (74.7)	64,733 (74.9)	179,459 (81.1)	<0.001	74,679 (80.0)	1938 (73.6)	14,781 (76.4)	57,960 (81.3)	<0.001	48,850 (70.0)	1441 (57.4)	17,414 (70.1)	29,995 (70.7)	<0.001	36,613 (70.4)	2466 (68.7)	8332 (69.2)	25,815 (70.9)	<0.001	28,456 (84.1)	810 (82.6)	7099 (80.2)	20,547 (85.7)	<0.001
Black	36,623 (11.2)	2612 (13.1)	12,161 (14.1)	21,850 (9.9)		10,662 (11.4)	377 (14.3)	3055 (15.8)	7230 (10.1)		8269 (11.9)	374 (14.9)	3564 (14.3)	4331 (10.2)		6455 (12.4)	468 (13.0)	1760 (14.6)	4227 (11.6)		3411 (10.1)	106 (10.8)	1255 (14.2)	2050 (8.5)	
Asian or Pacific Islander	27,799 (8.5)	2046 (10.2)	7976 (9.2)	17,777 (8.0)		7184 (7.7)	279 (10.6)	1278 (6.6)	5627 (7.9)		11,477 (16.5)	648 (25.8)	3356 (13.5)	7473 (17.6)		8227 (15.8)	576 (16.0)	1706 (14.2)	5945 (16.3)		1632 (4.8)	50 (5.1)	382 (4.3)	1200 (5.0)	
American Indian/Alaska Native	2503 (0.8)	240 (1.2)	810 (0.9)	1453 (0.7)		603 (0.6)	25 (0.9)	179 (0.9)	399 (0.6)		951 (1.4)	34 (1.4)	419 (1.7)	498 (1.2)		544 (1.0)	54 (1.5)	171 (1.4)	319 (0.9)		244 (0.7)	13 (1.3)	95 (1.1)	136 (0.6)	
Unknown	1735 (0.5)	157 (0.8)	707 (0.8)	871 (0.4)		182 (0.2)	14 (0.5)	49 (0.3)	119 (0.2)		210 (0.3)	14 (0.6)	84 (0.3)	112 (0.3)		185 (0.4)	25 (0.7)	63 (0.5)	97 (0.3)		79 (0.2)	2 (0.2)	25 (0.3)	52 (0.2)	
Grade (%)																									
Well differentiated; Grade I	27,658 (8.4)	1635 (8.2)	7758 (9.0)	18,265 (8.2)	<0.001	4168 (4.5)	315 (12.0)	1091 (5.6)	2762 (3.9)	<0.001	6382 (9.1)	266 (10.6)	2191 (8.8)	3925 (9.3)	<0.001	2042 (3.9)	85 (2.4)	376 (3.1)	1581 (4.3)	<0.001	1524 (4.5)	48 (4.9)	408 (4.6)	1068 (4.5)	0.019
Moderately differentiated; Grade II	197,211 (60.2)	11,703 (58.5)	53,183 (61.6)	132,325 (59.8)		11,799 (12.6)	401 (15.2)	2934 (15.2)	8464 (11.9)		10,147 (14.5)	437 (17.4)	3512 (14.1)	6198 (14.6)		11,084 (21.3)	339 (9.4)	2093 (17.4)	8652 (23.8)		10,876 (32.2)	327 (33.3)	2933 (33.1)	7616 (31.8)	
Poorly differentiated; Grade III	49,289 (15.0)	3595 (18.0)	12,008 (13.9)	33,686 (15.2)		11,917 (12.8)	336 (12.8)	2916 (15.1)	8665 (12.1)		6322 (9.1)	336 (13.4)	2102 (8.5)	3884 (9.2)		28,364 (54.5)	2429 (67.7)	7202 (59.9)	18,733 (51.5)		13,811 (40.8)	390 (39.8)	3647 (41.2)	9774 (40.8)	
Undifferentiated; anaplastic; Grade IV	6662 (2.0)	504 (2.5)	1582 (1.8)	4576 (2.1)		775 (0.8)	33 (1.3)	199 (1.0)	543 (0.8)		525 (0.8)	27 (1.1)	182 (0.7)	316 (0.7)		1014 (1.9)	82 (2.3)	248 (2.1)	684 (1.9)		506 (1.5)	16 (1.6)	136 (1.5)	354 (1.5)	
Unknown	46,980 (14.3)	2566 (12.8)	11,856 (13.7)	32,558 (14.7)		64,651 (69.3)	1548 (58.8)	12,202 (63.1)	50,901 (71.4)		46,381 (66.5)	1445 (57.5)	16,850 (67.8)	28,086 (66.2)		9520 (18.3)	654 (18.2)	2113 (17.6)	6753 (18.6)		7105 (21.0)	200 (20.4)	1732 (19.6)	5173 (21.6)	
SEER summary stage (%)																									
Localized	123,772 (37.8)	5541 (27.7)	31,756 (36.8)	86,475 (39.1)	<0.001	8322 (8.9)	361 (13.7)	1282 (6.6)	6679 (9.4)	<0.001	30,096 (43.1)	916 (36.5)	11,102 (44.7)	18,078 (42.6)	<0.001	11,323 (21.8)	432 (12.0)	1981 (16.5)	8910 (24.5)	<0.001	6658 (19.7)	103 (10.5)	1409 (15.9)	5146 (21.5)	<0.001
Regional	119,606 (36.5)	8359 (41.8)	31,834 (36.9)	79,413 (35.9)		26,766 (28.7)	721 (27.4)	5935 (30.7)	20,110 (28.2)		18,635 (26.7)	754 (30.0)	6897 (27.8)	10,984 (25.9)		15,619 (30.0)	989 (27.6)	3838 (31.9)	10,792 (29.6)		10,619 (31.4)	311 (31.7)	2814 (31.8)	7494 (31.2)	
Distant	70,053 (21.4)	5551 (27.8)	20,593 (23.8)	43,909 (19.8)		49,399 (52.9)	1425 (54.1)	11,276 (58.3)	36,698 (51.4)		12,341 (17.7)	619 (24.7)	4362 (17.6)	7360 (17.4)		19,828 (38.1)	1948 (54.3)	5539 (46.0)	12,341 (33.9)		12,905 (38.2)	493 (50.3)	4039 (45.6)	8373 (34.9)	
Unknown/unstaged	14,369 (4.4)	552 (2.8)	2204 (2.6)	11,613 (5.2)		8823 (9.5)	126 (4.8)	849 (4.4)	7848 (11.0)		8685 (12.5)	222 (8.8)	2476 (10.0)	5987 (14.1)		5254 (10.1)	220 (6.1)	674 (5.6)	4360 (12.0)		3640 (10.8)	74 (7.5)	594 (6.7)	2972 (12.4)	
T stage (%)																									
T0/Tis	12,109 (3.7)	553 (2.8)	3876 (4.5)	7680 (3.5)	<0.001	499 (0.5)	15 (0.6)	103 (0.5)	381 (0.5)	<0.001	97 (0.1)	4 (0.2)	32 (0.1)	61 (0.1)	<0.001	79 (0.2)	4 (0.1)	18 (0.1)	57 (0.2)	<0.001	18 (0.1)	0 (0.0)	4 (0.0)	14 (0.1)	<0.001
T1	53,727 (16.4)	2594 (13.0)	15,894 (18.4)	35,239 (15.9)		3098 (3.3)	126 (4.8)	645 (3.3)	2327 (3.3)		24,587 (35.2)	791 (31.5)	8646 (34.8)	15,150 (35.7)		12,080 (23.2)	577 (16.1)	2356 (19.6)	9147 (25.1)		9041 (26.7)	194 (19.8)	2225 (25.1)	6622 (27.6)	
T2	38,446 (11.7)	1867 (9.3)	9463 (11.0)	27,116 (12.2)		17,055 (18.3)	575 (21.8)	3354 (17.3)	13,126 (18.4)		12,376 (17.7)	388 (15.5)	5035 (20.3)	6953 (16.4)		14,012 (26.9)	936 (26.1)	3424 (28.5)	9652 (26.5)		2868 (8.5)	74 (7.5)	738 (8.3)	2056 (8.6)	
T3	144,721 (44.1)	9601 (48.0)	37,807 (43.8)	97,313 (44.0)		31,240 (33.5)	880 (33.4)	7136 (36.9)	23,224 (32.6)		14,595 (20.9)	638 (25.4)	5433 (21.9)	8524 (20.1)		6283 (12.1)	526 (14.7)	1652 (13.7)	4105 (11.3)		9111 (26.9)	281 (28.6)	2500 (28.2)	6330 (26.4)	
T4	45,863 (14.0)	3699 (18.5)	12,523 (14.5)	29,641 (13.4)		15,442 (16.5)	495 (18.8)	3861 (20.0)	11,086 (15.5)		3274 (4.7)	213 (8.5)	1095 (4.4)	1966 (4.6)		6148 (11.8)	587 (16.4)	1715 (14.3)	3846 (10.6)		4321 (12.8)	185 (18.9)	1398 (15.8)	2738 (11.4)	
Unknown	32,934 (10.0)	1689 (8.4)	6824 (7.9)	24,421 (11.0)		25,976 (27.8)	542 (20.6)	4243 (21.9)	21,191 (29.7)		14,828 (21.3)	477 (19.0)	4596 (18.5)	9755 (23.0)		13,422 (25.8)	959 (26.7)	2867 (23.8)	9596 (26.4)		8463 (25.0)	247 (25.2)	1991 (22.5)	6225 (26.0)	
N stage (%)																									
N0	184,507 (56.3)	8997 (45.0)	46,242 (53.5)	129,268 (58.4)	<0.001	42,628 (45.7)	1191 (45.2)	8278 (42.8)	33,159 (46.5)	<0.001	49,869 (71.5)	1756 (69.9)	18,231 (73.4)	29,882 (70.5)	<0.001	20,908 (40.2)	1207 (33.6)	4205 (34.9)	15,496 (42.6)	<0.001	13,651 (40.4)	301 (30.7)	3220 (36.4)	10,130 (42.2)	<0.001
≥N1	118,525 (36.2)	9774 (48.9)	35,108 (40.6)	73,643 (33.3)		28,130 (30.1)	983 (37.3)	7466 (38.6)	19,681 (27.6)		5760 (8.3)	318 (12.7)	2163 (8.7)	3279 (7.7)		21,739 (41.8)	1717 (47.8)	5875 (48.8)	14,147 (38.9)		15,057 (44.5)	549 (56.0)	4482 (50.6)	10,026 (41.8)	
Unknown	24,768 (7.6)	1232 (6.2)	5037 (5.8)	18,499 (8.4)		22,552 (24.2)	459 (17.4)	3598 (18.6)	18,495 (25.9)		14,128 (20.3)	437 (17.4)	4443 (17.9)	9248 (21.8)		9377 (18.0)	665 (18.5)	1952 (16.2)	6760 (18.6)		5114 (15.1)	131 (13.4)	1154 (13.0)	3829 (16.0)	
M stage (%)																									
M0	248,311 (75.8)	14,157 (70.8)	64,532 (74.7)	169,622 (76.6)	<0.001	38,264 (41.0)	1192 (45.3)	7905 (40.9)	29,167 (40.9)	<0.001	48,304 (69.2)	1633 (65.0)	17,819 (71.7)	28,852 (68.0)	<0.001	28,905 (55.6)	1504 (41.9)	6119 (50.9)	21,282 (58.5)	<0.001	19,438 (57.5)	478 (48.7)	4679 (52.8)	14,281 (59.5)	<0.001
M1	66,465 (20.3)	5292 (26.5)	19,578 (22.7)	41,595 (18.8)		46,059 (49.4)	1312 (49.8)	10,493 (54.2)	34,254 (48.0)		11,980 (17.2)	598 (23.8)	4242 (17.1)	7140 (16.8)		18,898 (36.3)	1888 (52.6)	5285 (43.9)	11,725 (32.2)		11,554 (34.2)	452 (46.1)	3665 (41.4)	7437 (31.0)	
Unknown	13,024 (4.0)	554 (2.8)	2277 (2.6)	10,193 (4.6)		8987 (9.6)	129 (4.9)	944 (4.9)	7914 (11.1)		9473 (13.6)	280 (11.2)	2776 (11.2)	6417 (15.1)		4221 (8.1)	197 (5.5)	628 (5.2)	3396 (9.3)		2830 (8.4)	51 (5.2)	512 (5.8)	2267 (9.5)	
Treatment (%)																									
No/unknown therapy	31,314 (9.6)	795 (4.0)	4391 (5.1)	26,128 (11.8)	<0.001	43,811 (47.0)	583 (22.1)	5805 (30.0)	37,423 (52.5)	<0.001	32,064 (46.0)	885 (35.2)	10,425 (42.0)	20,754 (48.9)	<0.001	14,739 (28.3)	599 (16.7)	2274 (18.9)	11,866 (32.6)	<0.001	8195 (24.2)	167 (17.0)	1551 (17.5)	6477 (27.0)	<0.001
Surgery only	166,636 (50.8)	6165 (30.8)	35,515 (41.1)	124,956 (56.4)		6060 (6.5)	487 (18.5)	1369 (7.1)	4204 (5.9)		9678 (13.9)	494 (19.7)	3680 (14.8)	5504 (13.0)		11,202 (21.5)	410 (11.4)	1829 (15.2)	8963 (24.6)		2948 (8.7)	57 (5.8)	709 (8.0)	2182 (9.1)	
Chemotherapy only	13,701 (4.2)	1278 (6.4)	4779 (5.5)	7644 (3.5)		25,344 (27.2)	882 (33.5)	7084 (36.6)	17,378 (24.4)		18,334 (26.3)	665 (26.5)	6997 (28.2)	10,672 (25.2)		9064 (17.4)	1152 (32.1)	2943 (24.5)	4969 (13.6)		4042 (12.0)	214 (21.8)	1313 (14.8)	2515 (10.5)	
Radiation only	1791 (0.5)	58 (0.3)	330 (0.4)	1403 (0.6)		1389 (1.5)	25 (0.9)	265 (1.4)	1099 (1.5)		2067 (3.0)	44 (1.8)	633 (2.5)	1390 (3.3)		1334 (2.6)	54 (1.5)	224 (1.9)	1056 (2.9)		2607 (7.7)	31 (3.2)	514 (5.8)	2062 (8.6)	
Combined surgery and chemotherapy	70,043 (21.4)	7182 (35.9)	24,542 (28.4)	38,319 (17.3)		5153 (5.5)	223 (8.5)	1401 (7.2)	3529 (4.9)		4916 (7.0)	276 (11.0)	2165 (8.7)	2475 (5.8)		4423 (8.5)	496 (13.8)	1373 (11.4)	2554 (7.0)		616 (1.8)	23 (2.3)	217 (2.5)	376 (1.6)	
Combined surgery and radiation	2247 (0.7)	119 (0.6)	560 (0.6)	1568 (0.7)		288 (0.3)	15 (0.6)	65 (0.3)	208 (0.3)		262 (0.4)	11 (0.4)	83 (0.3)	168 (0.4)		454 (0.9)	27 (0.8)	110 (0.9)	317 (0.9)		173 (0.5)	3 (0.3)	44 (0.5)	126 (0.5)	
Combined radiation and chemotherapy	8349 (2.5)	703 (3.5)	2737 (3.2)	4909 (2.2)		6654 (7.1)	241 (9.2)	1854 (9.6)	4559 (6.4)		2037 (2.9)	88 (3.5)	706 (2.8)	1243 (2.9)		4073 (7.8)	241 (6.7)	1029 (8.6)	2803 (7.7)		10,491 (31.0)	271 (27.6)	2799 (31.6)	7421 (30.9)	
Combined surgery, radiation and chemotherapy	33,719 (10.3)	3703 (18.5)	13,533 (15.7)	16,483 (7.4)		4611 (4.9)	177 (6.7)	1499 (7.7)	2935 (4.1)		399 (0.6)	48 (1.9)	148 (0.6)	203 (0.5)		6735 (12.9)	610 (17.0)	2250 (18.7)	3875 (10.6)		4750 (14.0)	215 (21.9)	1709 (19.3)	2826 (11.8)	
Marital status (%)																									
Divorced/separated	33,318 (10.2)	1604 (8.0)	10,779 (12.5)	20,935 (9.5)	<0.001	10,116 (10.8)	244 (9.3)	2827 (14.6)	7045 (9.9)	<0.001	9558 (13.7)	148 (5.9)	4205 (16.9)	5205 (12.3)	<0.001	4736 (9.1)	240 (6.7)	1465 (12.2)	3031 (8.3)	<0.001	4081 (12.1)	89 (9.1)	1354 (15.3)	2638 (11.0)	<0.001
Married	172,094 (52.5)	10,993 (55.0)	50,388 (58.3)	110,713 (50.0)		49,037 (52.6)	1347 (51.2)	10,931 (56.5)	36,759 (51.5)		34,916 (50.1)	1094 (43.6)	11,725 (47.2)	22,097 (52.1)		28,771 (55.3)	1946 (54.2)	7142 (59.4)	19,683 (54.1)		18,149 (53.7)	466 (47.5)	4490 (50.7)	13,193 (55.0)	
Single/unmarried	49,197 (15.0)	6361 (31.8)	18,096 (20.9)	24,740 (11.2)		12,435 (13.3)	917 (34.8)	4228 (21.9)	7290 (10.2)		14,232 (20.4)	1131 (45.0)	6987 (28.1)	6114 (14.4)		7659 (14.7)	1210 (33.7)	2570 (21.4)	3879 (10.7)		5761 (17.0)	387 (39.4)	2365 (26.7)	3009 (12.5)	
Widowed/others	73,191 (22.3)	1045 (5.2)	7124 (8.2)	65,022 (29.4)		21,722 (23.3)	125 (4.7)	1356 (7.0)	20,241 (28.4)		11,051 (15.8)	138 (5.5)	1920 (7.7)	8993 (21.2)		10,858 (20.9)	193 (5.4)	855 (7.1)	9810 (26.9)		5831 (17.2)	39 (4.0)	647 (7.3)	5145 (21.5)	
Income (%)																									
≤$64,999	162,453 (49.6)	9476 (47.4)	43,095 (49.9)	109,882 (49.6)	<0.001	44,784 (48.0)	1302 (49.4)	9629 (49.8)	33,853 (47.5)	<0.001	33,569 (48.1)	1122 (44.7)	12,240 (49.3)	20,207 (47.6)	<0.001	24,722 (47.5)	1673 (46.6)	5858 (48.7)	17,191 (47.2)	0.011	16,879 (49.9)	497 (50.7)	4639 (52.4)	11,743 (49.0)	<0.001
>$64,999	165,347 (50.4)	10,527 (52.6)	43,292 (50.1)	111,528 (50.4)		48,526 (52.0)	1331 (50.6)	9713 (50.2)	37,482 (52.5)		36,188 (51.9)	1389 (55.3)	12,597 (50.7)	22,202 (52.4)		27,302 (52.5)	1916 (53.4)	6174 (51.3)	19,212 (52.8)		16,943 (50.1)	484 (49.3)	4217 (47.6)	12,242 (51.0)	
Survival months (median [IQR])	58.0 [17.0, 106.0]	69.0 [29.0, 121.0]	73.0 [32.0, 123.0]	52.0 [12.0, 98.0]	<0.001	4.0 [1.0, 13.0]	13.0 [4.0, 53.0]	8.0 [2.0, 18.0]	4.0 [1.0, 11.0]	<0.001	8.0 [2.0, 31.0]	12.0 [2.0, 53.0]	10.0 [2.0, 37.0]	7.0 [1.0, 26.0]	<0.001	10.0 [3.0, 37.0]	11.0 [4.0, 37.0]	13.0 [4.0, 46.0]	9.0 [2.0, 35.0]	<0.001	10.0 [3.0, 30.0]	12.0 [5.0, 35.0]	12.0 [4.0, 35.0]	9.0 [3.0, 27.0]	<0.001

Abbreviation: SEER, Surveillance, Epidemiology, and End Results.

### 3.2 Time trends of CVD‐related mortality by age at diagnosis

**FIGURE 2:**
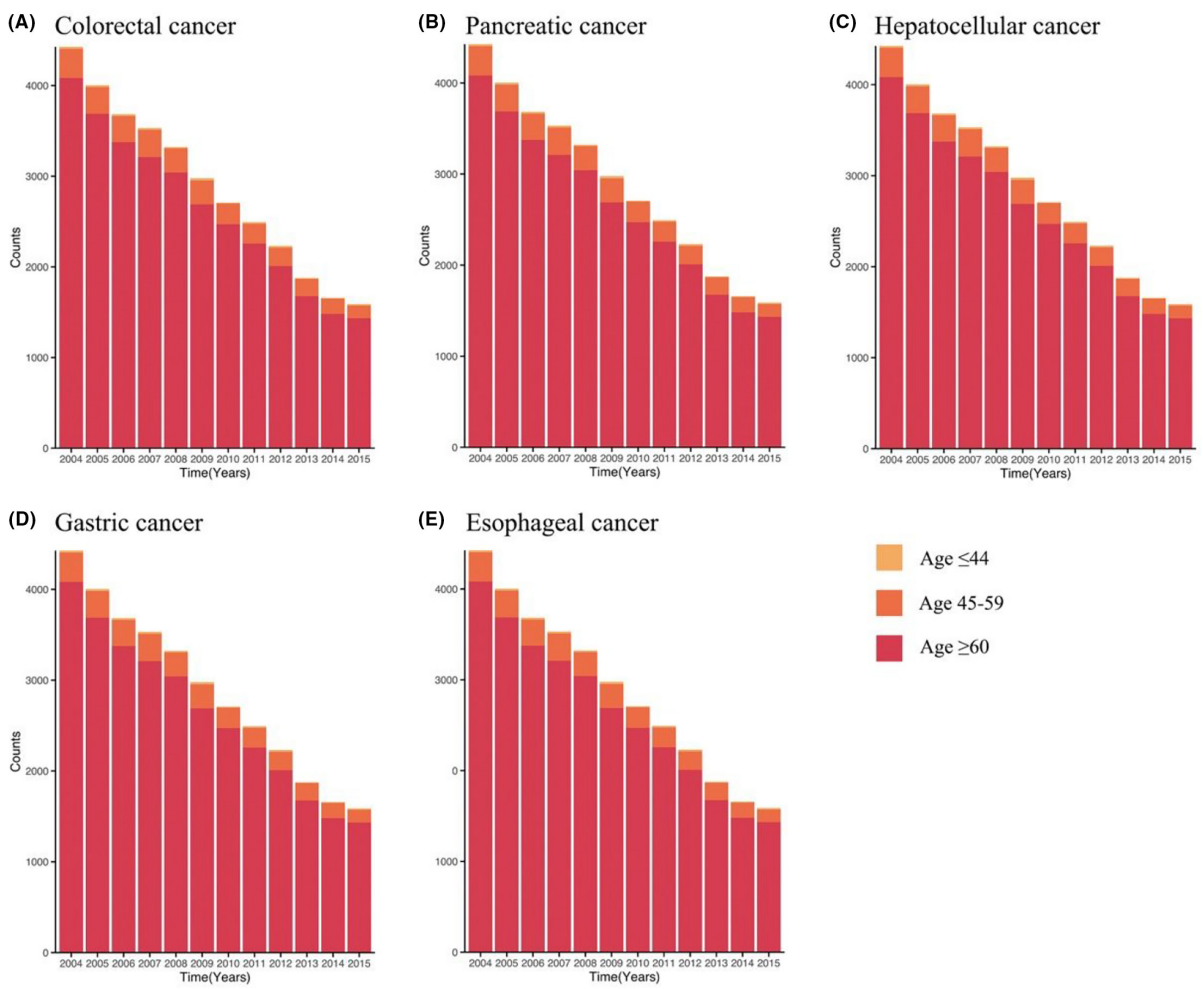
Distribution of age‐specific CVD‐related death among major gastrointestinal cancers diagnosed from 2004 to 2015 in the United States SEER population. (A) colorectal cancer, (B) pancreatic cancer, (C) hepatocellular cancer, (D) gastric cancer, and (E) esophageal cancer.

Between 2004 and 2015, a total of 26,512 (8.8%) colorectal cancer patients died from CVD in the SEER dataset; this number was 2027 (2.2%), 1961 (2.9%), 2508 (5.1%), and 1489 (4.6%), for among pancreatic cancer, hepatocellular cancer, gastric cancer, and esophageal cancer, respectively (Table S2). As depicted in Figure 2, since 2004, there has been a steady decline in the number of CVD‐related mortality, regardless of cancer type, and as illustrated in Figure S1, older patients (age at diagnosis ≥60 years) contributed to the most CVD‐related mortalities among the major gastrointestinal cancers.

### 3.3 CVD‐related mortality compared to the general population

**FIGURE 3:**
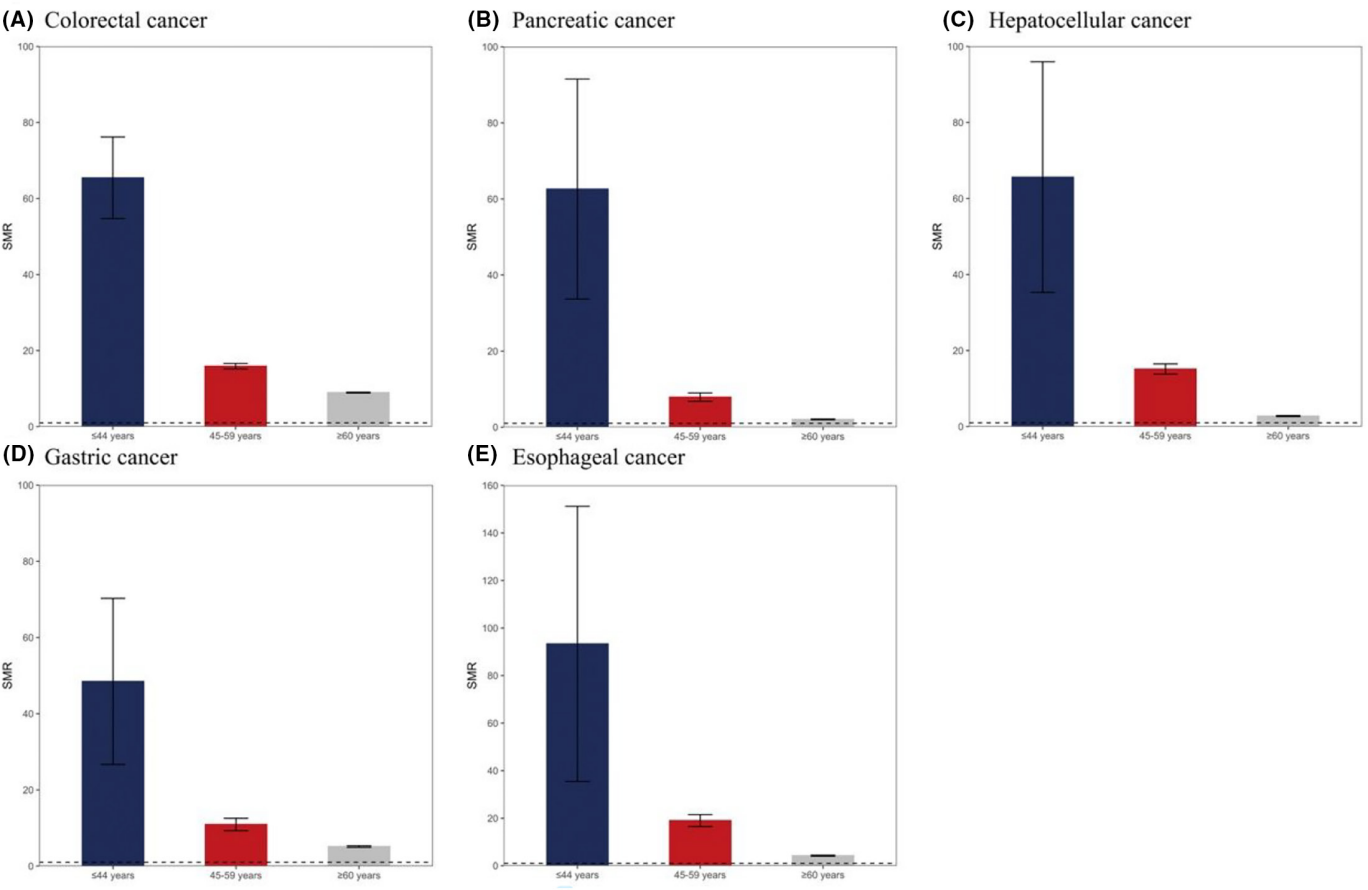
The bar chart of standardized mortality ratio (SMR) among major gastrointestinal cancers: (A) colorectal cancer, (B) pancreatic cancer, (C) hepatocellular cancer, (D) gastric cancer, and (E) esophageal cancer. The upper line presents the upper 95% confidence interval (CI), and the lower line means the lower 95% CI. The dashed line was set at 1.0 as a reference.

This study compared the age‐specific risks of CVD‐related mortality among the major causes of gastrointestinal cancer to the general U.S. population. At every age category (Figure 3, and Table S3), patients with gastrointestinal cancer experience excess CVD‐related mortality than the general population, independent of cancer type. Especially, the young group (age at diagnosis ≤44 years) had multifold higher risks, and the SMRs of the group among colorectal cancer, pancreatic cancer, hepatocellular cancer, gastric cancer, and esophageal cancer was 65.5 (95% CI: 54.7–76.2), 62.6 (95% CI: 33.7–91.5), 65.6 (95% CI: 35.3–96.0), 48.5 (95% CI: 26.7–70.3), and 93.3 (95% CI: 35.5–151.2), respectively.

### 3.4 Survival analysis and cumulative incidence of CVD

**FIGURE 4:**
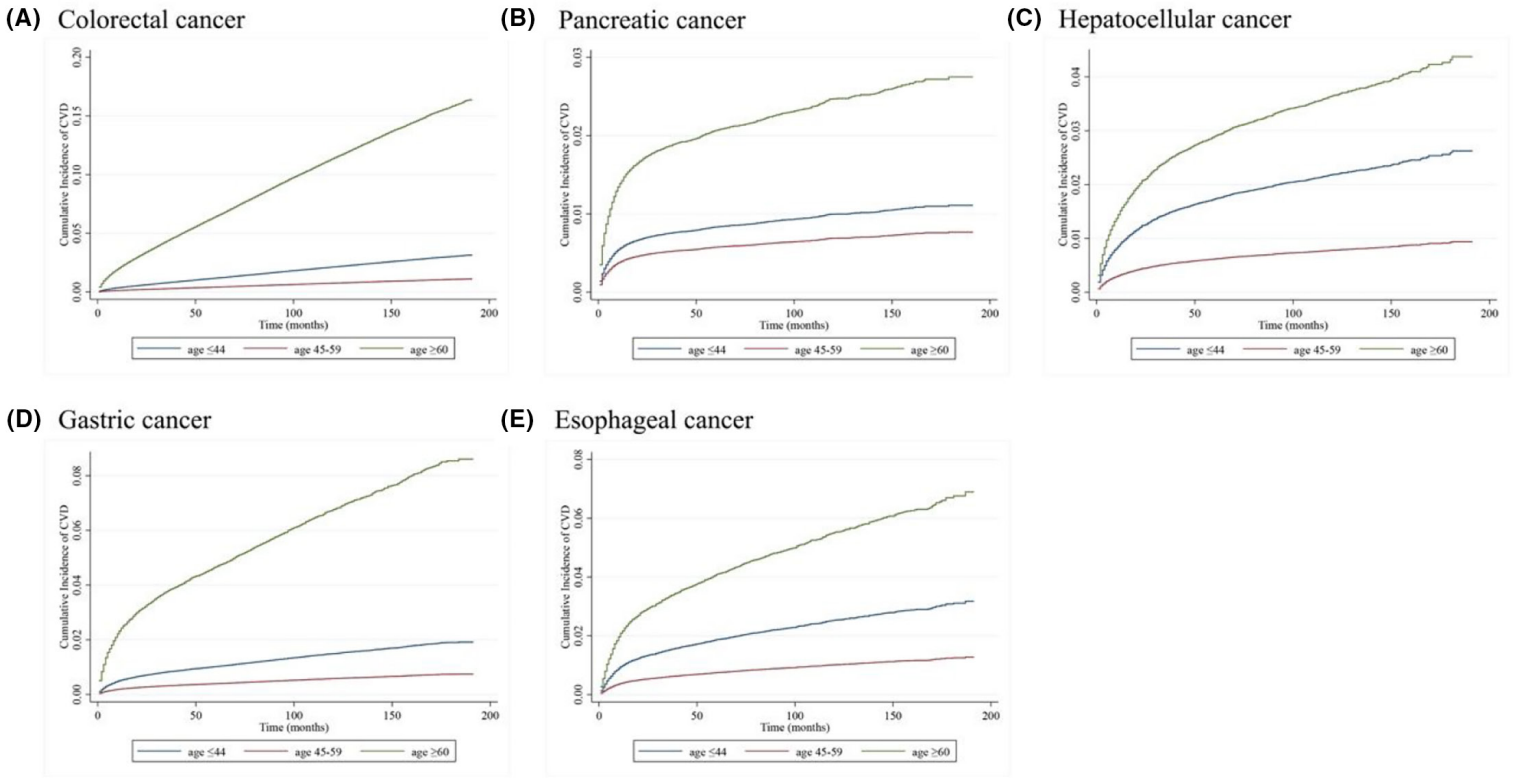
Cumulative incidence curve of CVD by different age at diagnosis among major gastrointestinal cancers: (A) colorectal cancer, (B) pancreatic cancer, (C) hepatocellular cancer, (D) gastric cancer, and (E) esophageal cancer.

The overall median follow‐up time was 24.0 (IQR: 4.0–79.0) months overall. As presented in Table 1, the median follow‐up time among patients with colorectal cancer was 58.0 (IQR: 17.0–106.0) months; there was a statistical difference between groups after age stratum: those young patients (age at diagnosis ≤44 years) were 69.0 (IQR: 29.0–121.0) months, those middle‐aged patients were 73.0 (IQR: 32.0–123.0) months, and those older patients were 52.0 (IQR: 12.0–98.0) months. Likewise, among patients with pancreatic cancer, the median follows of 4.0 (IQR: 1.0–13.0) months overall, 13.0 (IQR: 4.0–53.0) months for the young group, 8.0 (IQR: 2.0–18.0) months for the middle‐aged group, and 4.0 (IQR: 1.0–11.0) months for the older group. Among hepatocellular cancer patients, the median follow‐up was 8.0 (IQR: 2.0–31.0) months overall, 12.0 (IQR: 2.0–53.0) months for the young group, 10.0 (IQR: 2.0–37.0) months for the median‐aged group, and 7.0 (IQR: 1.0–26.0) months for the older group. Among gastric cancer patients, the median follow‐up was10.0 (IQR: 3.0–37.0) months overall, 11.0 (IQR: 4.0–37.0) months for the young group, 13.0 (IQR: 4.0–46.0) months for the middle‐aged group, and 9.0 (IQR: 2.0–35.0) months for the older group. Among esophageal cancer patients, the median follow‐up was 10.0 (IQR: 3.0–30.0) months overall, 12.0 (IQR: 5.0–35.0) months for the young group, 12.0 (IQR: 4.0–35.0) months for the middle‐aged group, and 9.0 (IQR: 3.0–27.0) months for the older group. As shown in Figure 4 (the cumulative incidence of CVD curve), the older patients (age at diagnosis ≥60 years) had a higher cumulative incidence of CVD than the young or middle‐aged patients.

### 3.5 Risk of CVD‐related mortality attributable to different groups of age at diagnosis

Our study assessed factors associated with CVD‐related mortality using univariate and multivariate competing risk regression analysis (Tables S4, S5, and 2). As presented in Table 2, among colorectal cancer patients, the crude SHRs for the middle‐aged and older groups were 0.19 (95% CI: 0.18–0.20) and 6.39 (95% CI: 6.10–6.70), respectively. Among pancreatic cancer patients, the SHRs for the middle‐aged and older groups were 0.41 (95% CI: 0.35–0.49) and 2.60 (95% CI: 2.22–3.03), respectively. Among hepatocellular cancer patients, the crude SHRs for the middle‐aged and older groups wre 0.62 (95% CI: 0.56–0.70) and 1.79 (95% CI: 1.61–1.99), respectively. Among gastric cancer patients, the crude SHRs for the middle‐aged and older groups were 0.24 (95% CI: 0.20–0.28) and 5.41 (95% CI: 4.64–6.31), respectively. Among esophageal cancer patients, the crude SHRs for the middle‐aged and older groups were 0.47 (95% CI: 0.40–0.54) and 2.36 (95% CI: 2.04–2.73), respectively. After adjusting for covariates noted in Table S5, the adjusted SHRs for the middle‐aged group among colorectal cancer, pancreatic cancer, hepatocellular cancer, gastric cancer, and esophageal cancer were 2.55 (95% CI: 2.15–3.03), 1.77 (95% CI: 1.06–2.97), 2.64 (95% CI: 1.60–4.36), 2.15 (95% CI: 1.32–3.51), and 2.28 (95% CI: 1.17–4.44), respectively. The adjusted SHRs for the older group among colorectal cancer, pancreatic cancer, hepatocellular cancer, gastric cancer, and esophageal cancer were 11.23 (95% CI: 9.50–13.27), 4.05 (95% CI: 2.46–6.66), 4.47 (95% CI: 2.72–7.35), 7.16 (95% CI: 4.49–11.41), and 4.40 (95% CI: 2.28–8.48), respectively.

**TABLE 2:** The sub‐hazards ratio of age categories for cardiovascular disease‐related mortality among major gastrointestinal cancers.

		Age		
		≤44	45–59	≥60
Colorectal cancer	SHR	1.00 [Reference]	0.19 (0.18–0.20)	6.39 (6.10–6.70)
	Adjusted SHR	1.00 [Reference]	2.55 (2.15–3.03)	11.23 (9.50–13.27)
Pancreatic cancer	SHR	1.00 [Reference]	0.41 (0.35–0.49)	2.60 (2.22–3.03)
	Adjusted SHR	1.00 [Reference]	1.77 (1.06–2.97)	4.05 (2.46–6.66)
Hepatocellular cancer	SHR	1.00 [Reference]	0.62 (0.56–0.70)	1.79 (1.61–1.99)
	Adjusted SHR	1.00 [Reference]	2.64 (1.60–4.36)	4.47 (2.72–7.35)
Gastric cancer	SHR	1.00 [Reference]	0.24 (0.20–0.28)	5.41 (4.64–6.31)
	Adjusted SHR	1.00 [Reference]	2.15 (1.32–3.51)	7.16 (4.49–11.41)
Esophageal cancer	SHR	1.00 [Reference]	0.47 (0.40–0.54)	2.36 (2.04–2.73)
	Adjusted SHR	1.00 [Reference]	2.28 (1.17–4.44)	4.40 (2.28–8.48)

Abbreviation: SHR, Sub‐hazards ratio.

### 3.6 Association between age at diagnosis (continuous) and CVD‐related mortality

**FIGURE 5:**
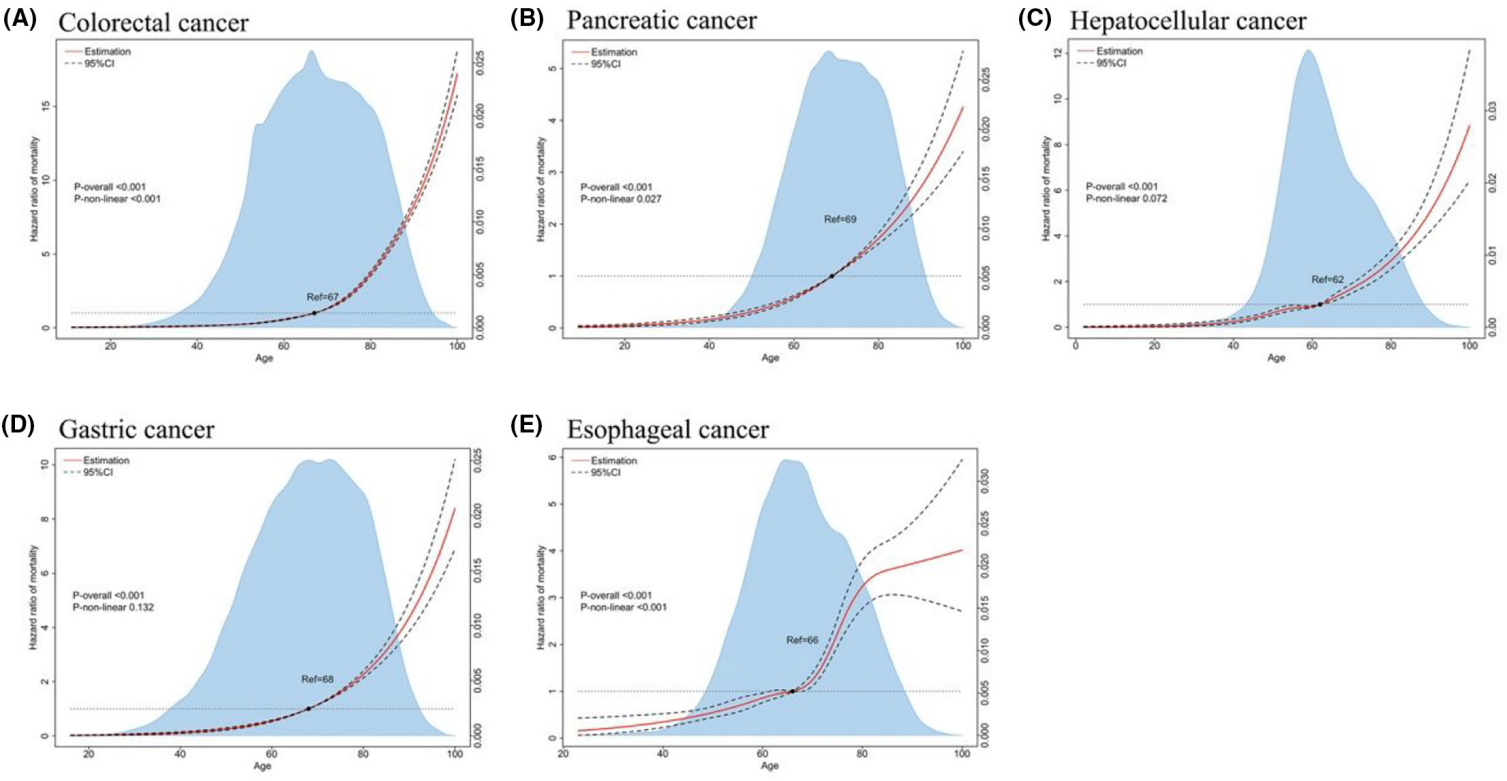
Risk of age‐specific CVD‐related mortality among major gastrointestinal cancers in the model of adjusted restricted cubic spline analyses: (A) colorectal cancer, (B) pancreatic cancer, (C) hepatocellular cancer, (D) gastric cancer, and (E) esophageal cancer. Solid red lines represented hazard ratios (HR) which were adjusted by multivariable. Dashed lines show 95% confidence intervals based on the restricted cubic spline regressions. The reference line for no association (hazard ratio: 1.0) is depicted by a dashed gray line while the reference value of age is based on it. Areas of blue show a fraction of the population at different ages.

To describe the dose–response analysis of age and CVD‐related mortality, we performed unadjusted and adjusted restricted cubic spline (RCS) analyses were performed in our study (Figures 5 and S2). The number of knots for the RCS model is listed in Table S6. In an unadjusted model, our study uncovered that expect for pancreatic cancer, other four major gastrointestinal cancers (colorectal cancer, hepatocellular cancer, gastric cancer, and esophageal cancer) had a non‐linear association between age at diagnosis and CVD‐related mortality (Figure S2). After adjusting confounders, a non‐linear relationship between age at diagnosis and CVD‐related mortality was found in colorectal cancer, pancreatic cancer, and esophageal cancer (Figure 5). The risk of CVD‐related mortality was higher when the age at diagnosis was over 67, 69, and 66 years old in colorectal cancer, pancreatic cancer, and esophageal cancer, respectively.

## 4 DISCUSSION

Cardio‐oncology is proposed as a new subspecialty to address the cardiovascular needs of cancer patients from diagnosis to treatment.^18^, ^19^, ^20^ It is recently introduced but rapidly evolving because of the undesirable consequences of aggressive anticancer therapies, for example, pericarditis, cardiomyopathies, and valvular dysfunction in the first year and the addition of coronary artery disease in the later decades.^21^, ^22^, ^23^ Prior studies have focused on CVD‐related mortality among cancer patients to increase public awareness and focus on CVD‐related mortality. For instance, Sun et al. reported that heart diseases accounted for approximately 5.3% of all deaths, second only to primary cancer in non‐small cell lung cancer, and the hazard ratio was gradually increasing with increasing age.^24^ Ramin et al. revealed that breast cancer survivors experienced a higher risk of CVD‐related mortality than cancer‐free women, with the highest risk among older survivors.^25^ Notably, age appears to be a risk factor for CVD‐related mortality among those cancer patients. However, studies have treated age as a categorical variable, or continuous variable, ignoring its non‐linear likelihood with CVD‐related mortality, which is unreasonable. A few studies have focused on the association between age and CVD‐related mortality in cancer patients, particularly for major gastrointestinal cancers. Moreover, among those major gastrointestinal cancers, there does not appear to be an age threshold at which the risk of CVD‐related death is negligible. Therefore, the present study investigated the association between age and CVD‐related mortality among patients with major gastrointestinal cancers with a large sample size.

A population‐based study of CVD‐related mortality risk in U.S. cancer patients demonstrated that increasing age at diagnosis was associated with an increased percentage of cancer patients dying from CVD, and cancer patients were perpetually at an elevated risk of dying from CVD when compared with the general U.S. population, with the risk decreasing with age at diagnosis.^26^ Similar to a previous study, our study observed that CVD events had a descending trend between 2004 and 2015 and that age at diagnosis ≥60 years accounted for the largest percentage of CVD‐related mortality. About the SMR, our study also found that CVD‐related mortality was greater among patients with major gastrointestinal cancers compared with the general U.S. population in all age strata, and young patients were associated with higher SMR, which is consistent with previous studies.^27^ One of the possible explanations for this phenomenon is that these patients shared risk factors that predisposing them to cancer and CVD.^28^, ^29^ Another is the cardio‐toxic side effects of those aggressive treatments existed.^26^, ^29^, ^30^ The assessment of SMR in our study presented significant preliminary population‐level data to support the development of clinical screening tools that could identify cancer patients at elevated risk for elevated CVD‐related mortality.

Studies have demonstrated that 16% of colorectal cancer patients aged 20–80 years old developed new‐onset CVD within 3 years of diagnosis,^31^ and among the older population (80% were aged ≥70 years old), 22.7% developed new‐onset CVD at 3 years after diagnosis and 56.7% at 10 years after diagnosis.^32^ A further study reported greater hazards among older patients for predicting post‐cancer CVD‐related morbidities, such as congestive heart failure (CHF) and CVD^32^. Regarding the cardiac mortality among those colorectal cancer patients, the study reported a 1.12‐fold increased risk of CVD for every 1‐year increase in age.^15^ In line with these studies, our study also found that middle‐aged (aged 45–59 years) and older (aged ≥60 years) colorectal cancer patients had a higher SHR than young patients (aged ≤44 years) for CVD‐related mortality. Besides, our study revealed a non‐linear association between age at diagnosis and CVD‐related mortality, and the risk of CVD‐related mortality was higher when the age at diagnosis was more than 67 years in the colorectal cancer population. There is a limited literature pertaining to pancreatic cancer, and hepatocellular cancer patients. A previous study found that patients with pancreatic cancer and hepatocellular cancer had a 1.10‐ and 1.08‐fold increased the risk of CVD for every 1‐year increase in age, respectively.^15^ Consistent with the previous studies, the middle‐aged and older groups were at a higher risk of CVD‐related mortality, and the older group had a higher cumulative incidence of CVD among pancreatic and hepatocellular cancer in our study. Moreover, our study highlighted the non‐linear association between age and CVD‐related mortality in pancreatic cancer patients and the linear association in hepatocellular cancer patients. The reference age at diagnosis in pancreatic cancer patients was 69 years. In gastric cancer patients, the 8‐year heart‐specific mortality sequentially increased with age; for instance, the mortality in older patients (aged ≥80 years) was about eight times that in those aged <60 years who underwent resection (and chemotherapy).^33^ The SHRs for heart‐specific mortality in older patients (aged ≥80 years) with resection alone and combined resection and chemotherapy treatment, respectively, were 3.88‐ and 2.72‐fold higher than those aged 60–69 years.^33^ Another study suggested that the SHR increased by 1.11‐fold for every 1‐year increase in age.^15^ Our study revealed that patients aged ≥60 years had a higher SHR value of 7.15 than patients with aged ≤44 years, and those older patients had a higher cumulative incidence of CVD. The RCS analyses validated that age at diagnosis was linearly associated with CVD mortality. Among esophageal cancer patients, age was regarded as the most risk factor determining heart death, with those aged ≥80 years being 14.297 times at risk for cardiac‐specific survival compared with those aged <40 years.^34^ Furthermore, the risk of cardiac mortality increased 1.08‐fold for every 1‐year increase in age.^15^ Similar to these studies, older esophageal cancer patients had a 4.40‐fold higher risk of CVD‐related mortality than those aged ≤44 years. Our study further found a non‐linear association between age at diagnosis and CVD‐related mortality. As esophageal cancer patients aged >66 years, the risk of a cardiac event was increases dramatically.

Our study found that (i) most patients with CVD‐related events were aged ≥60 years; (ii) there was a descending trend for CVD‐related death, and older patients make up the largest proportion of patients with CVD; (iii) all cancer patients exhibited with a higher SMR value than the general U.S. populations. Age at diagnosis was a negatively correlated with CVD‐related events; (iv) older patients (age at diagnosis ≥60 years) had a higher risk and cumulative incidence of CVD mortality compared with young patients (age at diagnosis ≤44 years); (v) age at diagnosis displayed a J‐shaped relationship with subsequent CVD events among colorectal cancer, pancreatic cancer, and esophageal cancer, and a linear relationship among hepatocellular cancer, and gastric cancer.

## 5 LIMITATIONS

Our study has some limitations. First, our retrospective study cannot compensate for all confounders, such as the history of medication. As we know, statin and anti‐platelet agents are the most common use in cardiovascular disease patients. Recent studies have revealed that statin and anti‐platelet agents could decrease the incidence of cancer,^35^, ^36^ which may influence the result. Second, the SEER database does not provide the baseline characteristics of CVD‐related comorbidity, contributing to inevitable biases, such as the tendency not to administer potentially cardiotoxic treatment to patients at risk of CVD at cancer diagnosis. Third, the miscoded CVD‐related death may arise from an overestimated heart disease in death certificate data.^37^

## 6 CONCLUSION

CVD‐related mortality gradually decreased every year among patients with major gastrointestinal cancers, and the majority were older patients. Compared with the general U.S. population, all cancer patients had higher CVD mortality. Furthermore, age was a risk factor for CVD mortality among these patients.

## AUTHOR CONTRIBUTIONS

**Gen Liu:** Conceptualization (equal); data curation (equal); formal analysis (equal); methodology (equal); software (equal); visualization (equal); writing – original draft (equal). **Bofang Zhang:** Funding acquisition (equal); investigation (equal); project administration (equal); resources (equal); supervision (equal); validation (equal); writing – review and editing (equal).

## CONFLICT OF INTEREST STATEMENT

The authors declare that they have no conflicts of interest in this paper.

## Supporting information


Table S1.

## Data Availability

Data sharing is not applicable to this article as no new data were created or analyzed in this study.
