# Supplementary material for: Age‐specific cardiovascular disease‐related mortality among patients with major gastrointestinal cancers: A SEER population‐based study
Source: Cancer Med. 2023 Jun 30;12(16):17253–65. doi: 10.1002/cam4.6305 (PMC10501270; doi:10.1002/cam4.6305)
Supplement: Supplementary file 1 — Table S1. [file CAM4-12-17253-s001.docx]

**Supplemental Materials**

**Supplemental Tables**

Table S1. The number and percentage of different cancers

Table S2. The number and percentage of cardiovascular-related outcomes among major gastrointestinal cancers

Table S3. SMRs of Mortality of Cardiovascular Diseases Among Different Cancer Types, by Age Category

Table S4. Univariate Competing Risk Regression Analysis of Factor Associated with Cardiovascular Mortality Among Different Cancer Types

Table S5. Multivariate Competing Risk Regression Analysis of Factor Associated with Cardiovascular Mortality Among Different Cancer Types

Table S6. Akaike information criterion value and related nodes among different gastrointestinal cancers

**Supplemental Figures**

Figure S1. Distribution of age-specific CVD-related death among major gastrointestinal cancers diagnosed from 2004 to 2015 in the United States SEER population

Figure S2. Risk of age-specific CVD-related mortality among major gastrointestinal cancers in the model of unadjusted restricted cubic spline analyse

Table S1. The number and percentage of different cancers

| The cancer type | The number of cancer cases | Percentage (%) |
| --- | --- | --- |
| Colon and Rectum | 327800 | 54.8 |
| Pancreas | 93310 | 15.6 |
| Liver and Intrahepatic bile duct | 69757 | 11.7 |
| Stomach | 52024 | 8.7 |
| Esophagus | 33822 | 5.7 |
| Anus, Anal Canal and Anorectum | 13781 | 2.3 |
| Small Intestine | 7207 | 1.2 |

Table S2. The number and percentage of cardiovascular-related outcomes among major gastrointestinal cancers

| Time(year) | Age | Colorectal cancer (N=301288) | |  | Pancreatic cancer (N=91283) | |  | Hepatocellular cancer (N=67796) | |  | Gastric cancer (N=49516) | |  | Esophageal cancer (N=32333) | |
| --- | --- | --- | --- | --- | --- | --- | --- | --- | --- | --- | --- | --- | --- | --- | --- |
|  |  | Counts (n=26512) | Percent(%) |  | Counts (n=2027) | Percent(%) |  | Counts (n=1961) | Percent(%) |  | Counts (n=2508) | Percent(%) |  | Counts (n=1489) | Percent(%) |
| 2004 | ≤44 | 16 | 0.43 |  | 0 | 0.00 |  | 2 | 1.74 |  | 1 | 0.33 |  | 2 | 1.33 |
| 2004 | 45-59 | 236 | 6.39 |  | 12 | 7.19 |  | 30 | 26.09 |  | 21 | 6.95 |  | 24 | 16.00 |
| 2004 | ≥60 | 3440 | 93.17 |  | 155 | 92.81 |  | 83 | 72.17 |  | 280 | 92.72 |  | 124 | 82.67 |
| 2005 | ≤44 | 16 | 0.49 |  | 2 | 1.12 |  | 1 | 0.78 |  | 0 | 0.00 |  | 2 | 1.26 |
| 2005 | 45-59 | 204 | 6.24 |  | 13 | 7.26 |  | 34 | 26.36 |  | 17 | 6.42 |  | 26 | 16.35 |
| 2005 | ≥60 | 3051 | 93.27 |  | 164 | 91.62 |  | 94 | 72.87 |  | 248 | 93.58 |  | 131 | 82.39 |
| 2006 | ≤44 | 14 | 0.46 |  | 1 | 0.61 |  | 2 | 1.59 |  | 2 | 0.84 |  | 1 | 0.76 |
| 2006 | 45-59 | 199 | 6.58 |  | 19 | 11.66 |  | 37 | 29.37 |  | 14 | 5.86 |  | 19 | 14.50 |
| 2006 | ≥60 | 2811 | 92.96 |  | 143 | 87.73 |  | 87 | 69.05 |  | 223 | 93.31 |  | 111 | 84.73 |
| 2007 | ≤44 | 19 | 0.68 |  | 2 | 1.04 |  | 0 | 0.00 |  | 1 | 0.41 |  | 0 | 0.00 |
| 2007 | 45-59 | 187 | 6.69 |  | 23 | 11.92 |  | 53 | 33.33 |  | 21 | 8.64 |  | 16 | 11.43 |
| 2007 | ≥60 | 2591 | 92.63 |  | 168 | 87.05 |  | 106 | 66.67 |  | 221 | 90.95 |  | 124 | 88.57 |
| 2008 | ≤44 | 11 | 0.42 |  | 1 | 0.60 |  | 0 | 0.00 |  | 1 | 0.42 |  | 3 | 2.38 |
| 2008 | 45-59 | 169 | 6.48 |  | 10 | 5.95 |  | 45 | 25.28 |  | 13 | 5.42 |  | 28 | 22.22 |
| 2008 | ≥60 | 2430 | 93.10 |  | 157 | 93.45 |  | 133 | 74.72 |  | 226 | 94.17 |  | 95 | 75.40 |
| 2009 | ≤44 | 13 | 0.59 |  | 5 | 2.56 |  | 5 | 2.55 |  | 1 | 0.45 |  | 0 | 0.00 |
| 2009 | 45-59 | 155 | 6.98 |  | 24 | 12.31 |  | 47 | 23.98 |  | 17 | 7.73 |  | 21 | 14.38 |
| 2009 | ≥60 | 2052 | 92.43 |  | 166 | 85.13 |  | 144 | 73.47 |  | 202 | 91.82 |  | 125 | 85.62 |
| 2010 | ≤44 | 6 | 0.30 |  | 1 | 0.56 |  | 0 | 0.00 |  | 3 | 1.43 |  | 0 | 0.00 |
| 2010 | 45-59 | 127 | 6.28 |  | 25 | 13.97 |  | 51 | 28.98 |  | 13 | 6.19 |  | 11 | 9.17 |
| 2010 | ≥60 | 1890 | 93.43 |  | 153 | 85.47 |  | 125 | 71.02 |  | 194 | 92.38 |  | 109 | 90.83 |
| 2011 | ≤44 | 13 | 0.71 |  | 0 | 0.00 |  | 3 | 1.74 |  | 1 | 0.55 |  | 0 | 0.00 |
| 2011 | 45-59 | 139 | 7.62 |  | 18 | 9.78 |  | 34 | 19.77 |  | 9 | 4.95 |  | 18 | 13.85 |
| 2011 | ≥60 | 1672 | 91.67 |  | 166 | 90.22 |  | 135 | 78.49 |  | 172 | 94.51 |  | 112 | 86.15 |
| 2012 | ≤44 | 12 | 0.75 |  | 2 | 1.22 |  | 2 | 1.09 |  | 3 | 1.63 |  | 1 | 0.95 |
| 2012 | 45-59 | 110 | 6.90 |  | 17 | 10.37 |  | 50 | 27.17 |  | 10 | 5.43 |  | 15 | 14.29 |
| 2012 | ≥60 | 1472 | 92.35 |  | 145 | 88.41 |  | 132 | 71.74 |  | 171 | 92.93 |  | 89 | 84.76 |
| 2013 | ≤44 | 5 | 0.38 |  | 2 | 1.47 |  | 1 | 0.65 |  | 3 | 1.75 |  | 0 | 0.00 |
| 2013 | 45-59 | 108 | 8.14 |  | 12 | 8.82 |  | 46 | 29.68 |  | 11 | 6.43 |  | 14 | 15.91 |
| 2013 | ≥60 | 1214 | 91.48 |  | 122 | 89.71 |  | 108 | 69.68 |  | 157 | 91.81 |  | 74 | 84.09 |
| 2014 | ≤44 | 6 | 0.55 |  | 1 | 0.62 |  | 1 | 0.54 |  | 1 | 0.83 |  | 1 | 1.03 |
| 2014 | 45-59 | 92 | 8.40 |  | 15 | 9.26 |  | 33 | 17.93 |  | 14 | 11.67 |  | 13 | 13.40 |
| 2014 | ≥60 | 997 | 91.05 |  | 146 | 90.12 |  | 150 | 81.52 |  | 105 | 87.50 |  | 83 | 85.57 |
| 2015 | ≤44 | 12 | 1.16 |  | 1 | 0.73 |  | 1 | 0.53 |  | 2 | 1.52 |  | 0 | 0.00 |
| 2015 | 45-59 | 69 | 6.67 |  | 12 | 8.76 |  | 32 | 17.11 |  | 12 | 9.09 |  | 15 | 15.46 |
| 2015 | ≥60 | 954 | 92.17 |  | 124 | 90.51 |  | 154 | 82.35 |  | 118 | 89.39 |  | 82 | 84.54 |

Table S3. SMRs of Mortality of Cardiovascular Diseases Among Different Cancer Types, by Age Category

| Cancer Types | Age Category^a^ | Observed deaths | Number of cancer cases | Crude risk of cardiovascular diseases^b^ in U.S. population (per 100,000) | Expected deaths^c^ | SMR (95%CI) |
| --- | --- | --- | --- | --- | --- | --- |
| Colorectal cancer | ≤44 years | 143 | 20003 | 11 | 2 | 65.5 (54.7-76.2) |
|  | 45-59 years | 1795 | 86387 | 131 | 113 | 15.9 (15.2-16.6) |
|  | ≥60 years | 24574 | 221410 | 1239 | 2743 | 9.0 (8.8-9.1) |
|  |  |  |  |  |  |  |
| Pancreatic cancer | ≤44 years | 18 | 2633 | 11 | 0 | 62.6 (33.7-91.5) |
|  | 45-59 years | 200 | 19342 | 131 | 25 | 7.9 (6.8-9.0) |
|  | ≥60 years | 1809 | 71335 | 1239 | 884 | 2.0 (2.0-2.1) |
|  |  |  |  |  |  |  |
| Hepatocellular cancer | ≤44 years | 18 | 2511 | 11 | 0 | 65.6 (35.3-96.0) |
|  | 45-59 years | 492 | 24837 | 131 | 32 | 15.2 (13.8-16.5) |
|  | ≥60 years | 1451 | 42409 | 1239 | 525 | 2.8 (2.6-2.9) |
|  |  |  |  |  |  |  |
| Gastric cancer | ≤44 years | 19 | 3589 | 11 | 0 | 48.5 (26.7-70.3) |
|  | 45-59 years | 172 | 12032 | 131 | 16 | 10.9 (9.3-12.6) |
|  | ≥60 years | 2317 | 36403 | 1239 | 451 | 5.1 (4.9-5.3) |
|  |  |  |  |  |  |  |
| Esophageal cancer | ≤44 years | 10 | 981 | 11 | 0 | 93.3 (35.5-151.2) |
|  | 45-59 years | 220 | 8856 | 131 | 12 | 19.0 (16.5-21.5) |
|  | ≥60 years | 1259 | 23985 | 1239 | 297 | 4.2 (4.0-4.5) |

^a^Age at cancer diagnosis or age in the general population

^b^Cardiovascular diseases in the U.S. population includes: diseases of heart (ICD 10 = I00-I02, I05-I09, I11, I13, I20-I25, I26-I28, I30-I51), hypertension without heart disease (ICD 10 = I10,I12), cerebrovascular diseases (ICD 10 = I60-I69), diseases of arteries, arterioles and capillaries (ICD 10 = I70-I78)

^c^Expected deaths equals to the age-stratum-specific crude risk of cardiovascular diseases in the U.S. population times to the age-stratum-specific number of cancer cases among different cancer types

Table S4 Univariate Competing Risk Regression Analysis of Factor Associated with Cardiovascular Mortality Among Different Cancer Types

|  | Colorectal cance (n=327800) | |  | Pancreatic cancer (n=93310) | |  | Hepatocellular cancer (n=69757) | |  | Gastric cancer (n=52024) | |  | Esophagus (n=33822) | |
| --- | --- | --- | --- | --- | --- | --- | --- | --- | --- | --- | --- | --- | --- | --- |
| Variable | SHR (95%CI) | P value |  | SHR (95%CI) | P value |  | SHR (95%CI) | P value |  | SHR (95%CI) | P value |  | SHR (95%CI) | P value |
| Sex (%) |  |  |  |  |  |  |  |  |  |  |  |  |  |  |
| Female | 1.00 [Reference] | NA |  | 1.00 [Reference] | NA |  | 1.00 [Reference] | NA |  | 1.00 [Reference] | NA |  | 1.00 [Reference] | NA |
| Male | 0.99 (0.97-1.02) | 0.637 |  | 0.96 (0.87-1.06) | 0.463 |  | 1.06 (0.95-1.18) | 0.291 |  | 0.92 (0.84-1.00) | 0.044 |  | 0.95 (0.83-1.08) | 0.415 |
| Race (%) |  |  |  |  |  |  |  |  |  |  |  |  |  |  |
| White | 1.00 [Reference] | NA |  | 1.00 [Reference] | NA |  | 1.00 [Reference] | NA |  | 1.00 [Reference] | NA |  | 1.00 [Reference] | NA |
| Black | 0.86 (0.83-0.90) | <0.001 |  | 1.41 (1.23-1.62) | <0.001 |  | 1.17 (1.02-1.35) | 0.026 |  | 1.16 (1.02-1.30) | 0.019 |  | 0.91 (0.76-1.10) | 0.333 |
| Asian or Pacific Islander | 0.69 (0.65-0.72) | <0.001 |  | 0.97 (0.80-1.17) | 0.754 |  | 0.92 (0.80-1.05) | 0.199 |  | 0.99 (0.88-1.10) | 0.795 |  | 0.89 (0.68-1.16) | 0.376 |
| American Indian/Alaska Native | 0.59 (0.49-0.71) | <0.001 |  | 0.73 (0.35-1.54) | 0.407 |  | 0.56 (0.33-0.97) | 0.039 |  | 0.65 (0.39-1.08) | 0.098 |  | 0.30 (0.10-0.94) | 0.038 |
| Unknown | 0.15 (0.09-0.24) | <0.001 |  | 0.80 (0.20-3.21) | 0.753 |  | 0.00 (0.00-0.00) | <0.001 |  | 0.35 (0.09-1.39) | 0.136 |  | 1.09 (0.35-3.41) | 0.881 |
| Grade (%) |  |  |  |  |  |  |  |  |  |  |  |  |  |  |
| Well differentiated; Grade I | 1.00 [Reference] | NA |  | 1.00 [Reference] | NA |  | 1.00 [Reference] | NA |  | 1.00 [Reference] | NA |  | 1.00 [Reference] | NA |
| Moderately differentiated; Grade II | 1.14 (1.11-1.17) | <0.001 |  | 1.26 (1.10-1.43) | 0.001 |  | 1.18 (1.04-1.33) | 0.01 |  | 1.58 (1.44-1.73) | <0.001 |  | 1.13 (1.01-1.26) | 0.037 |
| Poorly differentiated; Grade III | 0.89 (0.86-0.92) | <0.001 |  | 0.80 (0.69-0.94) | 0.007 |  | 0.81 (0.67-0.97) | 0.022 |  | 0.64 (0.59-0.70) | <0.001 |  | 0.69 (0.62-0.77) | <0.001 |
| Undifferentiated; anaplastic; Grade IV | 0.88 (0.80-0.97) | 0.007 |  | 0.61 (0.31-1.23) | 0.167 |  | 0.60 (0.28-1.26) | 0.176 |  | 0.86 (0.63-1.19) | 0.371 |  | 1.03 (0.67-1.58) | 0.893 |
| Unknown | 0.77 (0.74-0.81) | <0.001 |  | 0.89 (0.80-0.99) | 0.027 |  | 0.90 (0.81-0.99) | 0.03 |  | 0.93 (0.83-1.04) | 0.211 |  | 1.26 (1.12-1.43) | <0.001 |
| SEER summary stage (%) |  |  |  |  |  |  |  |  |  |  |  |  |  |  |
| Localized | 1.00 [Reference] | NA |  | 1.00 [Reference] | NA |  | 1.00 [Reference] | NA |  | 1.00 [Reference] | NA |  | 1.00 [Reference] | NA |
| Regional | 1.00 (0.97-1.03) | 0.98 |  | 1.18 (1.06-1.31) | 0.002 |  | 0.72 (0.64-0.81) | <0.001 |  | 0.90 (0.83-0.99) | 0.027 |  | 1.00 (0.89-1.12) | 0.976 |
| Distant | 0.25 (0.24-0.27) | <0.001 |  | 0.48 (0.43-0.54) | <0.001 |  | 0.46 (0.38-0.55) | <0.001 |  | 0.23 (0.21-0.27) | <0.001 |  | 0.35 (0.30-0.40) | <0.001 |
| Unknown/unstaged | 1.20 (1.13-1.28) | <0.001 |  | 1.50 (1.29-1.75) | <0.001 |  | 0.90 (0.77-1.06) | 0.197 |  | 1.61 (1.43-1.82) | <0.001 |  | 1.49 (1.28-1.74) | <0.001 |
| Age at diagnosis |  |  |  |  |  |  |  |  |  |  |  |  |  |  |
| ≤44 | 1.00 [Reference] | NA |  | 1.00 [Reference] | NA |  | 1.00 [Reference] | NA |  | 1.00 [Reference] | NA |  | 1.00 [Reference] | NA |
| 44-59 | 0.19 (0.18-0.20) | <0.001 |  | 0.41 (0.35-0.49) | <0.001 |  | 0.62 (0.56-0.70) | <0.001 |  | 0.24 (0.20-0.28) | <0.001 |  | 0.47 (0.40-0.54) | <0.001 |
| ≥60 | 6.39 (6.10-6.70) | <0.001 |  | 2.60 (2.22-3.03) | <0.001 |  | 1.79 (1.61-1.99) | <0.001 |  | 5.41 (4.64-6.31) | <0.001 |  | 2.36 (2.04-2.73) | <0.001 |
| T stage (%) |  |  |  |  |  |  |  |  |  |  |  |  |  |  |
| T0/Tis | 1.00 [Reference] | NA |  | 1.00 [Reference] | NA |  | 1.00 [Reference] | NA |  | 1.00 [Reference] | NA |  | 1.00 [Reference] | NA |
| T1 | 1.11 (1.08-1.15) | <0.001 |  | 2.13 (1.76-2.58) | <0.001 |  | 1.66 (1.50-1.82) | <0.001 |  | 2.04 (1.88-2.22) | <0.001 |  | 1.53 (1.37-1.71) | <0.001 |
| T2 | 1.45 (1.40-1.50) | <0.001 |  | 1.13 (1.00-1.28) | 0.052 |  | 0.97 (0.86-1.10) | 0.674 |  | 1.08 (0.99-1.18) | 0.091 |  | 1.32 (1.12-1.56) | 0.001 |
| T3 | 1.09 (1.06-1.12) | <0.001 |  | 1.02 (0.91-1.13) | 0.775 |  | 0.64 (0.56-0.73) | <0.001 |  | 0.64 (0.55-0.74) | <0.001 |  | 0.85 (0.75-0.96) | 0.009 |
| T4 | 0.55 (0.52-0.57) | <0.001 |  | 0.56 (0.48-0.66) | <0.001 |  | 0.66 (0.50-0.88) | 0.005 |  | 0.38 (0.31-0.46) | <0.001 |  | 0.53 (0.43-0.65) | <0.001 |
| Unknown | 0.66 (0.62-0.69) | <0.001 |  | 1.04 (0.93-1.17) | 0.468 |  | 0.74 (0.65-0.85) | <0.001 |  | 0.71 (0.64-0.80) | <0.001 |  | 0.83 (0.73-0.95) | 0.007 |
| N stage (%) |  |  |  |  |  |  |  |  |  |  |  |  |  |  |
| N0 | 1.00 [Reference] | NA |  | 1.00 [Reference] | NA |  | 1.00 [Reference] | NA |  | 1.00 [Reference] | NA |  | 1.00 [Reference] | NA |
| ≥N1 | 0.56 (0.55-0.58) | <0.001 |  | 0.75 (0.67-0.84) | <0.001 |  | 0.44 (0.34-0.57) | <0.001 |  | 0.54 (0.49-0.59) | <0.001 |  | 0.59 (0.53-0.66) | <0.001 |
| Unknown | 0.70 (0.66-0.75) | <0.001 |  | 1.03 (0.91-1.16) | 0.634 |  | 0.80 (0.70-0.92) | 0.001 |  | 0.81 (0.71-0.91) | 0.001 |  | 0.98 (0.84-1.15) | 0.824 |
| M stage (%) |  |  |  |  |  |  |  |  |  |  |  |  |  |  |
| M0 | 1.00 [Reference] | NA |  | 1.00 [Reference] | NA |  | 1.00 [Reference] | NA |  | 1.00 [Reference] | NA |  | 1.00 [Reference] | NA |
| M1 | 0.23 (0.22-0.25) | <0.001 |  | 0.49 (0.44-0.54) | <0.001 |  | 0.46 (0.38-0.55) | <0.001 |  | 0.22 (0.20-0.26) | <0.001 |  | 0.32 (0.27-0.37) | <0.001 |
| Unknown | 1.05 (0.98-1.12) | 0.161 |  | 1.39 (1.19-1.62) | <0.001 |  | 0.90 (0.77-1.05) | 0.171 |  | 1.51 (1.32-1.73) | <0.001 |  | 1.38 (1.16-1.65) | <0.001 |
| Treatment (%) |  |  |  |  |  |  |  |  |  |  |  |  |  |  |
| No/Unknown therapy | 1.00 [Reference] | NA |  | 1.00 [Reference] | NA |  | 1.00 [Reference] | NA |  | 1.00 [Reference] | NA |  | 1.00 [Reference] | NA |
| Surgery only | 2.63 (2.56-2.70) | <0.001 |  | 2.12 (1.83-2.44) | <0.001 |  | 1.49 (1.33-1.67) | <0.001 |  | 2.74 (2.52-2.98) | <0.001 |  | 1.90 (1.65-2.19) | <0.001 |
| Chemotherapy only | 0.19 (0.17-0.22) | <0.001 |  | 0.50 (0.44-0.57) | <0.001 |  | 0.80 (0.71-0.89) | <0.001 |  | 0.26 (0.22-0.31) | <0.001 |  | 0.37 (0.29-0.47) | <0.001 |
| Radiation only | 1.00 (0.84-1.19) | 0.993 |  | 0.99 (0.67-1.48) | 0.974 |  | 0.74 (0.54-1.01) | 0.055 |  | 0.77 (0.57-1.03) | 0.081 |  | 1.05 (0.87-1.28) | 0.603 |
| Combined surgery and chemotherapy | 0.38 (0.37-0.40) | <0.001 |  | 1.19 (0.99-1.43) | 0.071 |  | 0.98 (0.83-1.17) | 0.857 |  | 0.46 (0.37-0.56) | <0.001 |  | 0.73 (0.47-1.14) | 0.164 |
| Combined surgery and radiation | 1.01 (0.87-1.16) | 0.942 |  | 1.40 (0.70-2.80) | 0.339 |  | 1.09 (0.55-2.16) | 0.81 |  | 1.13 (0.76-1.67) | 0.542 |  | 1.19 (0.62-2.28) | 0.611 |
| Combined radiation and chemotherapy | 0.64 (0.58-0.71) | <0.001 |  | 0.61 (0.49-0.76) | <0.001 |  | 0.48 (0.33-0.70) | <0.001 |  | 0.82 (0.70-0.97) | 0.017 |  | 1.01 (0.90-1.13) | 0.915 |
| Combined surgery, radiation and chemotherapy | 0.49 (0.46-0.52) | <0.001 |  | 0.92 (0.74-1.15) | 0.475 |  | 0.26 (0.08-0.81) | 0.02 |  | 0.67 (0.58-0.77) | <0.001 |  | 0.95 (0.82-1.10) | 0.503 |
| Marital status (%) |  |  |  |  |  |  |  |  |  |  |  |  |  |  |
| Divorced/Separated | 1.00 [Reference] | NA |  | 1.00 [Reference] | NA |  | 1.00 [Reference] | NA |  | 1.00 [Reference] | NA |  | 1.00 [Reference] | NA |
| Married | 0.69 (0.67-0.70) | <0.001 |  | 0.77 (0.70-0.85) | <0.001 |  | 0.90 (0.82-0.99) | 0.03 |  | 0.70 (0.64-0.76) | <0.001 |  | 0.93 (0.83-1.03) | 0.154 |
| Single/Unmarried | 0.76 (0.73-0.79) | <0.001 |  | 0.94 (0.80-1.09) | 0.399 |  | 0.99 (0.88-1.12) | 0.845 |  | 0.73 (0.64-0.83) | <0.001 |  | 0.85 (0.73-0.99) | 0.034 |
| Widowed/Others | 2.08 (2.02-2.13) | <0.001 |  | 1.42 (1.27-1.59) | <0.001 |  | 1.33 (1.17-1.50) | <0.001 |  | 1.99 (1.82-2.18) | <0.001 |  | 1.46 (1.29-1.66) | <0.001 |
| Income (%) |  |  |  |  |  |  |  |  |  |  |  |  |  |  |
| ≤$64,999 | 1.00 [Reference] | NA |  | 1.00 [Reference] | NA |  | 1.00 [Reference] | NA |  | 1.00 [Reference] | NA |  | 1.00 [Reference] | NA |
| >$64,999 | 1.00 (0.98-1.03) | 0.707 |  | 0.95 (0.86-1.06) | 0.361 |  | 1.05 (0.96-1.16) | 0.281 |  | 0.96 (0.88-1.04) | 0.288 |  | 1.04 (0.93-1.16) | 0.482 |

Abbreviations: SHR, sub-hazard ratio; NA, not applicable; SEER, Surveillance, Epidemiology, and End Results.

Table S5. Multivariate Competing Risk Regression Analysis of Factor Associated with Cardiovascular Mortality Among Different Cancer Types

|  | Colorectal cance^a^ (n=327800) | |  | Pancreatic cancer^a^ (n=93310) | |  | Hepatocellular cancer^a^ (n=69757) | |  | Gastric cancer^a^ (n=52024) | |  | Esophagus^b^ (n=33822) | |
| --- | --- | --- | --- | --- | --- | --- | --- | --- | --- | --- | --- | --- | --- | --- |
| Variable | SHR (95%CI) | P value |  | SHR (95%CI) | P value |  | SHR (95%CI) | P value |  | SHR (95%CI) | P value |  | SHR (95%CI) | P value |
| Race (%) |  |  |  |  |  |  |  |  |  |  |  |  |  |  |
| White | 1.00 [Reference] | NA |  | 1.00 [Reference] | NA |  | 1.00 [Reference] | NA |  | 1.00 [Reference] | NA |  | 1.00 [Reference] | NA |
| Black | 0.96 (0.92-1.00) | 0.038 |  | 1.52 (1.32-1.76) | <0.001 |  | 1.19 (1.03-1.37) | 0.02 |  | 1.16 (1.03-1.31) | 0.018 |  | 0.97 (0.80-1.17) | 0.717 |
| Asian or Pacific Islander | 0.73 (0.69-0.77) | <0.001 |  | 1.02 (0.84-1.23) | 0.861 |  | 0.89 (0.77-1.02) | 0.089 |  | 0.88 (0.78-0.98) | 0.025 |  | 0.87 (0.67-1.14) | 0.309 |
| American Indian/Alaska Native | 0.66 (0.55-0.79) | <0.001 |  | 0.83 (0.40-1.74) | 0.624 |  | 0.58 (0.34-1.00) | 0.051 |  | 0.74 (0.44-1.23) | 0.247 |  | 0.33 (0.10-1.01) | 0.053 |
| Unknown | 0.14 (0.09-0.23) | <0.001 |  | 0.74 (0.18-2.95) | 0.667 |  | 0.00 (0.00-0.00) | <0.001 |  | 0.34 (0.08-1.36) | 0.126 |  | 0.91 (0.29-2.86) | 0.876 |
| Grade (%) |  |  |  |  |  |  |  |  |  |  |  |  |  |  |
| Well differentiated; Grade I | 1.00 [Reference] | NA |  | 1.00 [Reference] | NA |  | 1.00 [Reference] | NA |  | 1.00 [Reference] | NA |  | 1.00 [Reference] | NA |
| Moderately differentiated; Grade II | 0.99 (0.95-1.03) | 0.564 |  | 0.97 (0.77-1.21) | 0.762 |  | 0.98 (0.82-1.18) | 0.859 |  | 1.03 (0.87-1.22) | 0.698 |  | 0.93 (0.74-1.16) | 0.505 |
| Poorly differentiated; Grade III | 0.98 (0.93-1.03) | 0.399 |  | 0.75 (0.58-0.96) | 0.02 |  | 0.83 (0.66-1.04) | 0.109 |  | 0.84 (0.71-0.99) | 0.04 |  | 0.78 (0.62-0.99) | 0.04 |
| Undifferentiated; anaplastic; Grade IV | 0.96 (0.86-1.06) | 0.422 |  | 0.59 (0.29-1.21) | 0.152 |  | 0.58 (0.27-1.25) | 0.165 |  | 0.85 (0.60-1.20) | 0.36 |  | 0.99 (0.61-1.59) | 0.956 |
| Unknown | 0.91 (0.86-0.96) | 0.001 |  | 0.93 (0.75-1.16) | 0.525 |  | 0.93 (0.79-1.08) | 0.345 |  | 0.89 (0.74-1.08) | 0.242 |  | 1.02 (0.80-1.30) | 0.863 |
| SEER summary stage (%) |  |  |  |  |  |  |  |  |  |  |  |  |  |  |
| Localized | 1.00 [Reference] | NA |  | 1.00 [Reference] | NA |  | 1.00 [Reference] | NA |  | 1.00 [Reference] | NA |  | 1.00 [Reference] | NA |
| Regional | 1.04 (1.00-1.09) | 0.051 |  | 0.76 (0.60-0.96) | 0.02 |  | 0.79 (0.68-0.92) | 0.002 |  | 0.97 (0.80-1.17) | 0.741 |  | 0.65 (0.54-0.77) | <0.001 |
| Distant | 0.96 (0.82-1.12) | 0.61 |  | 0.67 (0.46-0.96) | 0.031 |  | 0.51 (0.24-1.09) | 0.083 |  | 0.92 (0.60-1.40) | 0.689 |  | 0.56 (0.40-0.79) | 0.001 |
| Unknown/unstaged | 1.23 (1.06-1.43) | 0.007 |  | 0.79 (0.58-1.09) | 0.152 |  | 0.96 (0.69-1.34) | 0.811 |  | 1.40 (1.04-1.89) | 0.029 |  | 0.89 (0.71-1.11) | 0.303 |
| Age at diagnosis |  |  |  |  |  |  |  |  |  |  |  |  |  |  |
| ≤44 | 1.00 [Reference] | NA |  | 1.00 [Reference] | NA |  | 1.00 [Reference] | NA |  | 1.00 [Reference] | NA |  | 1.00 [Reference] | NA |
| 44-59 | 2.55 (2.15-3.03) | <0.001 |  | 1.77 (1.06-2.97) | 0.03 |  | 2.64 (1.60-4.36) | <0.001 |  | 2.15 (1.32-3.51) | 0.002 |  | 2.28 (1.17-4.44) | 0.015 |
| ≥60 | 11.23 (9.50-13.27) | <0.001 |  | 4.05 (2.46-6.66) | <0.001 |  | 4.47 (2.72-7.35) | <0.001 |  | 7.16 (4.49-11.41) | <0.001 |  | 4.40 (2.28-8.48) | <0.001 |
| T stage (%) |  |  |  |  |  |  |  |  |  |  |  |  |  |  |
| T0/Tis | 1.00 [Reference] | NA |  | 1.00 [Reference] | NA |  | 1.00 [Reference] | NA |  | 1.00 [Reference] | NA |  | - | - |
| T1 | 1.04 (0.97-1.12) | 0.237 |  | 0.92 (0.42-2.02) | 0.842 |  | 1.40 (0.19-10.17) | 0.741 |  | 0.84 (0.20-3.49) | 0.814 |  | - | - |
| T2 | 1.22 (1.14-1.31) | <0.001 |  | 0.71 (0.33-1.51) | 0.368 |  | 1.17 (0.16-8.51) | 0.878 |  | 0.73 (0.18-3.04) | 0.669 |  | - | - |
| T3 | 1.19 (1.11-1.28) | <0.001 |  | 0.71 (0.33-1.50) | 0.368 |  | 0.99 (0.14-7.23) | 0.994 |  | 0.55 (0.13-2.30) | 0.414 |  | - | - |
| T4 | 0.89 (0.82-0.97) | 0.007 |  | 0.53 (0.25-1.14) | 0.102 |  | 1.22 (0.16-9.03) | 0.846 |  | 0.46 (0.11-1.92) | 0.285 |  | - | - |
| Unknown | 0.89 (0.77-1.03) | 0.13 |  | 0.71 (0.33-1.52) | 0.376 |  | 0.94 (0.13-6.85) | 0.955 |  | 0.56 (0.14-2.33) | 0.428 |  | - | - |
| N stage (%) |  |  |  |  |  |  |  |  |  |  |  |  |  |  |
| N0 | 1.00 [Reference] | NA |  | 1.00 [Reference] | NA |  | 1.00 [Reference] | NA |  | 1.00 [Reference] | NA |  | 1.00 [Reference] | NA |
| ≥N1 | 0.86 (0.83-0.90) | <0.001 |  | 0.85 (0.74-0.98) | 0.029 |  | 0.67 (0.51-0.88) | 0.004 |  | 0.80 (0.68-0.94) | 0.008 |  | 0.95 (0.80-1.12) | 0.517 |
| Unknown | 0.88 (0.79-0.97) | 0.013 |  | 1.01 (0.84-1.22) | 0.898 |  | 0.90 (0.73-1.11) | 0.34 |  | 0.80 (0.65-0.98) | 0.029 |  | 0.87 (0.68-1.11) | 0.27 |
| M stage (%) |  |  |  |  |  |  |  |  |  |  |  |  |  |  |
| M0 | 1.00 [Reference] | NA |  | 1.00 [Reference] | NA |  | 1.00 [Reference] | NA |  | 1.00 [Reference] | NA |  | 1.00 [Reference] | NA |
| M1 | 0.39 (0.33-0.46) | <0.001 |  | 0.68 (0.49-0.94) | 0.021 |  | 1.03 (0.49-2.19) | 0.93 |  | 0.45 (0.30-0.69) | <0.001 |  | 0.57 (0.41-0.79) | 0.001 |
| Unknown | 0.83 (0.75-0.92) | 0.001 |  | 0.86 (0.65-1.14) | 0.304 |  | 0.97 (0.75-1.24) | 0.784 |  | 1.14 (0.93-1.41) | 0.21 |  | 1.04 (0.79-1.36) | 0.781 |
| Treatment (%) |  |  |  |  |  |  |  |  |  |  |  |  |  |  |
| No/Unknown therapy | 1.00 [Reference] | NA |  | 1.00 [Reference] | NA |  | 1.00 [Reference] | NA |  | 1.00 [Reference] | NA |  | 1.00 [Reference] | NA |
| Surgery only | 1.05 (0.98-1.12) | 0.183 |  | 1.47 (1.21-1.79) | <0.001 |  | 1.06 (0.91-1.23) | 0.428 |  | 1.47 (1.29-1.68) | <0.001 |  | 1.27 (1.04-1.56) | 0.017 |
| Chemotherapy only | 0.51 (0.44-0.60) | <0.001 |  | 0.69 (0.59-0.79) | <0.001 |  | 0.81 (0.71-0.92) | 0.001 |  | 0.61 (0.50-0.75) | <0.001 |  | 0.71 (0.54-0.94) | 0.016 |
| Radiation only | 0.93 (0.78-1.12) | 0.472 |  | 0.83 (0.56-1.24) | 0.373 |  | 0.72 (0.52-0.98) | 0.04 |  | 0.76 (0.56-1.04) | 0.087 |  | 1.14 (0.91-1.43) | 0.261 |
| Combined surgery and chemotherapy | 0.53 (0.49-0.58) | <0.001 |  | 1.03 (0.81-1.30) | 0.816 |  | 0.85 (0.70-1.04) | 0.112 |  | 0.64 (0.51-0.81) | <0.001 |  | 0.88 (0.55-1.40) | 0.58 |
| Combined surgery and radiation | 0.84 (0.71-0.98) | 0.026 |  | 1.15 (0.57-2.33) | 0.702 |  | 0.87 (0.43-1.73) | 0.683 |  | 1.12 (0.74-1.68) | 0.603 |  | 1.18 (0.61-2.31) | 0.624 |
| Combined radiation and chemotherapy | 0.76 (0.68-0.85) | <0.001 |  | 0.58 (0.46-0.74) | <0.001 |  | 0.54 (0.37-0.80) | 0.002 |  | 0.98 (0.81-1.18) | 0.825 |  | 1.16 (0.99-1.37) | 0.074 |
| Combined surgery, radiation and chemotherapy | 0.55 (0.51-0.60) | <0.001 |  | 0.82 (0.63-1.07) | 0.142 |  | 0.27 (0.09-0.84) | 0.023 |  | 0.84 (0.70-1.01) | 0.06 |  | 1.09 (0.89-1.35) | 0.4 |
| Marital status (%) |  |  |  |  |  |  |  |  |  |  |  |  |  |  |
| Divorced/Separated | 1.00 [Reference] | NA |  | 1.00 [Reference] | NA |  | 1.00 [Reference] | NA |  | 1.00 [Reference] | NA |  | 1.00 [Reference] | NA |
| Married | 0.97 (0.93-1.02) | 0.289 |  | 0.84 (0.71-0.99) | 0.034 |  | 1.03 (0.89-1.20) | 0.683 |  | 0.88 (0.75-1.03) | 0.104 |  | 1.06 (0.88-1.27) | 0.523 |
| Single/Unmarried | 1.17 (1.10-1.24) | <0.001 |  | 0.93 (0.76-1.14) | 0.492 |  | 1.18 (0.99-1.40) | 0.064 |  | 0.95 (0.78-1.14) | 0.566 |  | 1.11 (0.90-1.39) | 0.327 |
| Widowed/Others | 1.46 (1.39-1.53) | <0.001 |  | 0.95 (0.80-1.14) | 0.591 |  | 1.24 (1.04-1.48) | 0.015 |  | 1.25 (1.06-1.46) | 0.007 |  | 1.25 (1.02-1.53) | 0.031 |

^a^In the model of multivariate cox regression, those variables of race, grade, SEER summary stage, age, T stage, N stage, M stage, treatment, and marital status were included.

^b^In the model of multivariate cox regression, those variables of race, grade, SEER summary stage, age, N stage, M stage, treatment, and marital status were included, and the variable of T stage was removed.

Abbreviations: SHR, sub-hazard ratio; NA, not applicable; SEER, Surveillance, Epidemiology, and End Results.

Table S6. Akaike information criterion value and related nodes among different gastrointestinal cancers

|  | Number of nodes | 3 | 4 | 5 | 6 | 7 |
| --- | --- | --- | --- | --- | --- | --- |
| Colorectal cancer | Unadjusted | 601989.9 | 601981.1 | 601973.1 | 601966.7 | **601961.2** |
|  | Adjusted | 599652.0 | 599615.4 | 599600.0 | 599590.5 | **599582.3** |
|  |  |  |  |  |  |  |
| Pancreatic cancer | Unadjusted | **41080.1** | 41081.8 | 41083.5 | 41085.5 | 41087.6 |
|  | Adjusted | **40617.0** | 40618.6 | 40620.6 | 40622.5 | 40623.9 |
|  |  |  |  |  |  |  |
| Hepatocellular cancer | Unadjusted | 38991.4 | 38986.5 | 38985.3 | **38984.7** | 38986.3 |
|  | Adjusted | 38489.7 | 38490.7 | 38487.1 | **38486.7** | 38488.1 |
|  |  |  |  |  |  |  |
| Gastric cancer | Unadjusted | **46649.4** | 46651.1 | 46653.2 | 46653.3 | 46651.4 |
|  | Adjusted | **46248.1** | 46249.7 | 46251.5 | 46251.7 | 46249.7 |
|  |  |  |  |  |  |  |
| Esophageal cancer | Unadjusted | 26837.5 | 26837.2 | 26822.3 | **26819.1** | 26822.6 |
|  | Adjusted | 26658.0 | 26651.9 | 26633.6 | **26630.1** | 26633.6 |

Figure S1. Distribution of age-specific CVD-related death among major gastrointestinal cancers diagnosed from 2004 to 2015 in the United States SEER population. (A) colorectal cancer, (B) pancreatic cancer, (C) hepatocellular cancer, (D) gastric cancer, and (E) esophageal cancer.

Figure S2. Risk of age-specific CVD-related mortality among major gastrointestinal cancers in the model of unadjusted restricted cubic spline analyses: (A) colorectal cancer, (B) pancreatic cancer, (C) hepatocellular cancer, (D) gastric cancer, and (E) esophageal cancer
